# Differential impact of *Paenibacillus* infection on the microbiota of *Varroa destructor* and *Apis mellifera*

**DOI:** 10.1016/j.heliyon.2024.e39384

**Published:** 2024-10-16

**Authors:** Štefánia Skičková, Karolína Svobodová, Apolline Maitre, Alejandra Wu-Chuang, Lianet Abuin-Denis, Elianne Piloto-Sardiñas, Dasiel Obregon, Igor Majláth, Viktória Majláthová, Alena Krejčí, Alejandro Cabezas-Cruz

**Affiliations:** a Pavol Jozef Šafárik University in Košice, Faculty of Science, Institute of Biology and Ecology, Institute of Animal Physiology, Košice, Slovakia; b University of South Bohemia, Faculty of Science, České Budějovice, Czech Republic; c ANSES, INRAE, École Nationale Vétérinaire d’Alfort, UMR BIPAR, Laboratoire de Santé Animale, Maisons-Alfort, F-94700, France; d EA 7310, Laboratoire de Virologie, Université de Corse, Corte, France; e INRAE, UR 0045, Laboratoire de Recherches Sur Le Développement de L'Élevage ‘(SELMET-LRDE), Corte, France; f Animal Biotechnology Department, Center for Genetic Engineering and Biotechnology, Avenue 31 Between 158 and 190, P.O. Box 6162, Havana, 10600, Cuba; g Direction of Animal Health, National Center for Animal and Plant Health, Carretera de Tapaste y Autopista Nacional, Apartado Postal 10, 32700, San José de las Lajas, Mayabeque, Cuba; h School of Environmental Sciences, University of Guelph, 50 Stone Rd E, Guelph, N1H 2W1, Ontario, Canada; i Czech Academy of Sciences, Biology Centre, Institute of Entomology, České Budějovice, 37005, Czech Republic

**Keywords:** *Paenibacillus* sp., *Varroa destructor*, *Apis mellifera*, Microbiota analysis, Microbial networks

## Abstract

The Western honey bee (*Apis mellifera*) is a vital agricultural pollinator whose populations are threatened by the parasitic mite *Varroa* destructor and associated pathogens. While the impact of *Paenibacillus* species on honey bees, particularly *Paenibacillus* larvae causing American foulbrood, is documented, their effect on the microbiota of *Varroa* mites remains unclear. This study aimed to investigate the influence of *Paenibacillus* sp. on the bacterial communities of *Varroa* mites and adult honey bees. We hypothesized that *Paenibacillus* sp. would significantly alter the microbiota of *Varroa* mites but have minimal effect on that of adult honey bees. Utilizing 16S rRNA sequencing data from a previous study, we reanalyzed samples categorized into four groups based on *Paenibacillus* sp. infection load: highly infected and lowly infected honey bees (*A. mellifera*) and mites (*V. destructor*). Infection status was determined by *Paenibacillus* sp. read counts, with more than three reads indicating high infection. Microbial diversity was assessed using alpha and beta diversity metrics. Co-occurrence networks were constructed to visualize bacterial community assemblies, and network robustness was evaluated through node addition and removal tests. Keystone taxa were identified based on eigenvector centrality and relative abundance. Highly infected *Varroa* mites exhibited a significant reduction in alpha diversity and a markedly different bacterial community composition compared to lowly infected mites (*p* < 0.05). Their bacterial co-occurrence networks showed decreased connectivity and robustness, indicating a disruptive effect of *Paenibacillus* sp. In contrast, adult honey bees displayed no significant differences in alpha diversity or network structure between highly and lowly infected groups (*p* > 0.05), suggesting a resilient microbiota. Keystone taxa analysis revealed fewer central species in highly infected *Varroa* mites, potentially impacting network stability. High *Paenibacillus* sp. infection is associated with significant alterations in the microbiota of *Varroa* mites, disrupting bacterial communities and potentially affecting mite physiology. The microbiota of adult honey bees appears more robust against *Paenibacillus* sp. influence. These findings enhance our understanding of the complex interactions within the “honey bee–mite–microorganism” system and may inform future strategies for managing *Varroa* mite infestations and associated pathogens.

## Highlights

• Study examines *Paenibacillus* impact on *Varroa* and bee microbiota.
• Varroa mites with *Paenibacillus* show reduced microbial diversity.
• *Paenibacillus* alters bacterial networks in *Varroa* mites significantly.

## 1 Introduction

Western honey bee (*Apis mellifera*) is an important agricultural pollinator contributing to global food production [1]. In recent decades, the widespread parasitic mite *Varroa destructor*, the major honey bee pest, has resulted in massive losses of honey bee colonies [2]. The main threat of the infestation by this parasite stems from its interactions with honey bee pathogens enhancing their deadly potential [3]. However, the impacts of honey bee pathogens on the biological aspects of *Varroa* mites are not fully elucidated. Unraveling the connections within the interconnected system “honey bee-mite-associated microorganisms” can yield valuable biological insights.

The gut microbiota of honey bees harbors relatively simple composition of bacterial species. The core bacteria phylotypes found in adult honey bee guts typically include *Lactobacillus* Firm-4 and Firm-5, *Snodgrassella alvei*, *Gilliamella apicola*, and *Frischella perara*, *Bifidobacterium* with additional bacterial taxa such as *Bartonella apis*, *Bombella apis,* and *Commensalibacter* are commonly present in adult honey bee samples [4]. These bacteria inhabit distinct parts of honey bee guts [5], cooperates with each other [6], and their activity is important for honey bee physiological processes. For instance, *Lactobacillus* strains provide metabolites involved in olfactory learning and memory [7], *Snodgrassella* is involved in decomposition of pectin, supporting effective digestion of pollen grains [8]. Also, gut microbiota plays an important role in the modulation of immune system, e.g. by stimulating production of antimicrobial peptides [9].

Several bacterial taxa have been associated with *Varroa* mites—namely, Enterobacteriaceae, *Pseudomonas*, *Enterococcus*, *Morganella*, and *Arsenophonus* [[10], [11], [12], [13]]— suggesting these bacteria as common inhabitants of the mite. Despite the *Varroa* mite's well-defined ecological niche as a highly specialized parasite of the honey bee, residing exclusively within honey bee hives and feeding on honey bee body components [14], detailed knowledge about the conservation and function of the bacteria associated with these mites is still lacking. While the relationships between the mite and associated bacteria remain largely unexplored, there has been a more detailed examination of its association with deadly honey bee pathogen, such as the Deformed Wing Virus (DWV) [14].

Building on this understanding, the study by Hubert et al. further explores the microbial interactions between *Varroa* mites and honey bees, identifying shared bacterial taxa that suggest a possible transfer of these bacteria between the parasite and its host [11]. For example, *Spiroplasma*, the agent of honey bee “May disease” [15], has been identified as abundant in *Varroa* mites [11]. Despite the potential for the presence and proliferation of honey bee pathogens to induce immune changes in the *Varroa* mite, as described in ticks and associated pathogens [16], their impact on *Varroa* mite physiology remains largely unclear.

Honey bee hives commonly contain several environmental bacteria, including species from the *Paenibacillus* genus [17]. Honey bees are associated with both pathogenic and non-pathogenic *Paenibacillus* species. Specifically, honey bees are known to interact with four key species: *Paenibacillus larvae*, which is the primary causative agent of American foulbrood disease [18,19], a devastating bacterial infection targeting honey bee larvae. Notably, *P. larvae* spores cannot germinate in adult honey bees, leaving this stage of the bee's life cycle unaffected [19]. Other species include *Paenibacillus melissococcoides*, a possible pathogen of honey bee larvae [20], *Paenibacillus apiarius* [21], and *Paenibacillus apis* [22], both found in bee colonies but not a significant pathogen, and *Paenibacillus intestini* [22], part of the bee gut microbiota. Several other *Paenibacillus* species, such as *Paenibacillus alvei* [23], *Paenibacillus dendriformis* [24], *Paenibacillus thiaminolyticus* [21,25] are primarily environmental bacteria found in the hive. These species are not typically harmful to bees [23,24,26,27], though *P. alvei* can act as a secondary invader in colonies affected by European foulbrood disease.

*Paenibacillus larvae* has also been associated with *Varroa* mite. In honey bee colonies highly affected by American foulbrood, the spores of *P. larvae* were found attached to the surface of *Varroa* mites [28,29]. Additionally, *in vitro* cultivated homogenate of *Varroa* mites from hives with American foulbrood resulted in the growth of *P. larvae* colonies, suggesting the presence of viable *P. larvae* bacteria inside the mite [29]. However, experimental inoculation of honey bee colonies with *P. larvae* through *Varroa* mites carrying a high number of spores did not lead to the outbreak of American foulbrood [28], thus suggesting that, under these experimental conditions, *Varroa* mites do not act as a vector for the disease. However, an unspecified *Paenibacillus* sp. has been identified to be involved in the bacterial community of *Varroa* mite [11], suggesting that *Paenibacillus* sp. possibly affects the assembly of bacterial community in the mites, but the exact impact of the bacterium on the bacterial association within the *Varroa* mites has not been shown.

This study was designed to explore the differential impact of *Paenibacillus* sp. on the bacterial communities of *Varroa* mites and adult honey bees. We hypothesized that *Paenibacillus* sp. significantly influences the microbiota of *Varroa* mites, potentially due to their direct feeding on honey bee fat body and hemolymph, which could introduce different microbial interactions than those occurring in the gut of nectar-feeding adult bees. Conversely, since *Paenibacillus* sp., including the pathogenic *P. larvae*, typically exists in a dormant spore form in adult bees that does not affect their immediate health, we proposed that these bacteria do not significantly alter the adult bees' bacterial community. This hypothesis addresses the observation that despite the known pathogenicity of *Paenibacillus* sp. in bee larvae, adult bees seem unaffected in terms of microbial diversity and function.

To test our hypothesis, we employed a comprehensive analysis of the 16S rRNA sequence dataset originally compiled by Hubert et al. [11]. This dataset included samples from *Varroa* mites and adult honey bees that were collected from colonies known to test positive for *P. larvae*. To specifically address the impact of *Paenibacillus* sp., we first categorized these samples into groups based on the quantitative presence of *Paenibacillus* reads. Samples were classified as either “highly infected” if they contained more than three reads of *Paenibacillus* sp., or “lowly infected” if they contained three or fewer, enabling a targeted exploration of bacterial community changes relative to the level of *Paenibacillus* infection. Utilizing an experimental network approach, we analyzed the topological differences in the bacterial networks between these defined groups. This methodological framework allowed us to identify patterns of microbial association and assess network resilience to perturbations, which provided insights into how *Paenibacillus* sp. might disrupt or stabilize bacterial communities differently in mites compared to bees. Furthermore, we identified specific bacterial taxa that correlated directly with the presence of *Paenibacillus* sp. and examined the impact of these correlations on the connectivity of two prominent bacterial taxa, *Escherichia* and *Rickettsiella*, within the microbiomes of the respective hosts. These findings validate our hypothesis regarding the differential impacts of *Paenibacillus* sp. on *Varroa* mites and honey bees, enhancing our understanding of the complex “honey bee–mite–microorganism” interactions and offering potential strategies for targeted intervention in managing Varroa mite infestations and associated pathogens effectively.

## 2 Methods

### 2.1 Original dataset

In this study, we analyzed available 16S rRNA datasets. The original study by Hubert et al. compared the microbial abundance and diversity in the microbiomes of *Apis mellifera* honey bee and *Varroa destructor* mite [11]. Samples were collected from seven apiaries in Czechia, resulting in 26 pairs of mites and adult worker honey bees. Prior to sampling, experimental colonies underwent acaricidal treatment and experimental *Varroa* mites were sampled from bottom boards. The DNA was isolated from whole-body homogenates of pooled honey bees and *Varroa* mites, which were surface-washed with ethanol prior to homogenization. The pools consisted of 10 adult worker honey bees and 10 to 50 female mites. The 16S rRNA gene fragments were PCR-amplified using 27Fmod/ill519Rmod barcoded primers targeting V1-V3 hypervariable regions and sequenced by Illumina TruSeq. The data was downloaded from NCBI GenBank under the SRA number SRP067076.

The dataset included two samples that were likely misclassified, with one labeled as a female honey bee that was a female *Varroa* and another labeled as a worker *Varroa* that was a honey bee. We corrected these misclassifications by reassigning each sample to its appropriate category before proceeding with the analysis. In addition, one sample of honey bee and one of *Varroa* mite were discarded from the analysis due to inaccuracies in their accession numbers, as they shared the same identifier.

### 2.2 Grouping based on *Paenibacillus* sp. infection load

To investigate the impact of *Paenibacillus* sp. on the microbial communities of honey bees and Varroa mites, we divided the dataset into four groups based on the *Paenibacillus* sp. read counts obtained from the 16S rRNA sequencing data. Samples with *Paenibacillus* sp. read counts of three or fewer were considered to have a low infection load, while those with more than three reads were considered highly infected. This threshold was chosen based on the distribution of *Paenibacillus* sp. read counts across samples, ensuring a clear distinction between low and high infection levels for comparative analysis. The four groups were AM-high (*A. mellifera*, highly infected, *n* = 15), AM-low (*A. mellifera*, lowly infected, *n* = 10), VD-high (*V. destructor*, highly infected, *n* = 17), and VD-low (*V. destructor*, lowly infected, *n* = 8) (Supplementary Table S1). This grouping allowed us to compare the microbial communities between highly and lowly infected honey bees and *Varroa* mites, facilitating the assessment of *Paenibacillus* sp.'s impact on their microbiota.

### 2.3 Bioinformatics analysis of 16S rRNA amplicon sequences

All sequencing processing was performed using Quantitative Insights Into Microbial Ecology (QIIME2) environment [30], version 2023.7. The original (.fastq) 16S rRNA sequences from the NCBI SRA were downloaded using q-fondue plugin [31]. Data were denoised, merged, and filtered using DADA2 method [32] implemented in QIIME2 [30], version 2023.7. Representative sequences were annotated using Bayes taxonomic classifier [33] based on 16S rRNA SILVA database (v138) [34] (Supplementary file S1).

### 2.4 Microbial diversity and relative abundance

Microbial diversity of bacterial community was analyzed applying alpha and beta diversities. Alpha diversity providing an intra-group analysis was measured by the metrics of Pielou's evenness [35], observed features [36], and Shannon entropy [37] using a Mann-Whitney test (*p* < 0.05) performed in GraphPad Prism 9.0.2. (GraphPad Software Inc., San Diego, CA, USA). Pielou's evenness index measures the evenness of taxa distribution in the groups. Observed features refer to the number of taxa or “features” observed in the groups describing the taxa richness. Shannon index measures the taxa diversity while taking into account both taxa richness and evenness. These methods were applied to evaluate the evenness, richness, and diversity of bacterial communities among several groups of honey bees and *Varroa* mites, both *Paenibacillus* highly and lowly infected. Beta diversity [38] identifying a between-group dissimilarity was carried out by Principal Coordinates Analysis (PCoA) plot based on Bray-Curtis distance matrix [39], PERMANOVA test (*p* < 0.05) in QIIME2 2023.7 (Supplementary file S1) [30], and ANOVA test (*p* < 0.05) with betadisper function [40] from Vegan package in R software 4.3.2 [41], performed using RStudio (Supplementary file S2) [42]. The PCoA method was applied to visualize the similarities or dissimilarities among the samples and analyze the community composition. Specifically, by applying this method, we aimed to compare the diversity of several groups included in this analysis to perform the evaluation of *Paenibacillus* infection. Cluster analysis was conducted using the Jaccard coefficient of similarity, implemented in Vegan package [40] in R 4.3.2 [41], performed using RStudio (Supplementary file S3) [42].

### 2.5 Unique and shared taxa identification

The quantification of unique and shared taxa was displayed by UpSet plot provided by UpSetR package [43], implemented in R 4.3.2 [41], and performed using RStudio (Supplementary file S4) [42]. The bacterial contribution abundance to highly and lowly infected groups was also displayed by Venn diagram performed by online tool available on website https://bioinformatics.psb.ugent.be/webtools/Venn/.

### 2.6 Bacterial co-occurrence networks

Co-occurrence network analysis was conducted using the Sparse Correlations for Computational data (SparCC) method [44], implemented in R 4.3.2 [41], and performed using RStudio (Supplementary file S5) [42]. Correlation coefficients of SparCC ≥0.5 or ≤ −0.5 were selected. Network visualization and calculation of topological features and taxa connectedness (i.e., number of nodes and edges, modularity, network diameter, average degree, weighted degree, clustering coefficient, and centrality metrics) were performed using the software Gephi 0.9.2 [45].

#### 2.6.1 Correlation analysis

Correlation analyses were performed on the topological features—betweenness, degree, and eigenvector centrality—of taxa present in networks with SparCC correlation coefficients of ≥0.3 or ≤ −0.3. These analyses were conducted using GraphPad Prism version 9.0.2 (GraphPad Software Inc., San Diego, CA, USA). The choice between Pearson and Spearman correlation methods was based on the distribution of the data as determined by the Shapiro-Wilk test for normality: Pearson correlation was used for normally distributed data, while Spearman correlation was applied for data that did not meet the normality assumption. Statistical significance was assessed accordingly.

#### 2.6.2 Core association networks

The Core Association Network (CAN) analysis refers to the networks of microbial taxa that are consistently associated with each other across different individuals or conditions. This analysis provides the insights into the interactions and relationships between different microbes within the microbiota. In this analysis, CAN was applied to both highly and lowly infected networks with SparCC ≥0.5 or ≤ −0.5. The analysis was conducted using Anuran toolbox [46] with default parameters in the Anaconda Python environment (Supplementary file S6) [47].

#### 2.6.3 Comparative network analysis

Various aspects of importance or centrality of individual nodes within the networks were analyzed using the Network construction and Comparison for Microbiome data (NetCoMi) package [48], implemented in R 4.3.2 [41], and performed using RStudio (Supplementary file S7) [42]. As correlations are calculated using the SpiecEasi method, the correlations found slightly differ from the co-occurrence networks generated using the SparCC algorithm. The analysis was performed to investigate the similarities or differences in the networks. The Jaccard index which calculates local centrality measures (i.e., degree, betweenness, closeness, eigenvector, and hub taxa) was employed to assess the similarity between the sets of the most central nodes. The Jaccard index measures the level of node similarity and ranges from 0 (totally different) to 1 (unique). The *p*-values, either *p* ≤ J or *p* ≥ J, correspond to “less than or equal” and “higher than or equal”, respectively, and represent the likelihood of Jaccard index deviating from the expected value. The Adjusted Rand Index (ARI) measures the dissimilarity between data clustering and ranges from −1 (different clustering) to 1 (identical). The negative values represent lower than random clustering while positive ones stand for higher than random clustering.

#### 2.6.4 Network robustness

The network robustness analysis measures the effects of node addition and removal on network connectivity. We stimulated these effects on the networks with SparCC ≥0.5 or ≤ - 0.5. Node addition analysis was performed using a method outlined by Freitas et al. [49], implemented in R 4.3.2 [41], and performed using RStudio (Supplementary file S8) [42]. New nodes in the number of 1000 were randomly added to the network and their impact was evaluated by Average Path Length (APL) and Largest Connected Component (LCC). The resulting average values were visualized by GraphPad Prism 9.0.2 (GraphPad Software Inc., San Diego, CA, USA).

To evaluate network robustness to node removal, we employed both random and directed attacks. For directed attack, three methods were applied: degree centrality, betweenness centrality, and cascading. In degree centrality approach, nodes with the highest degree centrality values were sequentially removed. In betweenness centrality approach, nodes with the highest betweenness centrality values were prioritized for removal. The cascading method involved initial removal of nodes with the highest betweenness centrality values, followed by a recalculation of betweenness centrality after each removal to dynamically adjust the subsequent removals. For the analysis of network robustness, the Network Strengths and Weaknesses Analysis (NetSwan) package [50] was used within the RStudio environment (Supplementary file S9) [42].

### 2.7 Keystone taxa identification

Keystone taxa were identified based on three established criteria [51]: (i) presence across all samples within an experimental group (ubiquitousness), (ii) eigenvector centrality exceeding 0.75, and (iii) mean higher than the average relative abundance compared to the average relative abundance across all taxa in the experimental group. Eigenvector centrality quantifies an importance of a node within a network where a higher score indicates strong connections with other highly scored nodes. Eigenvector centrality values were obtained using Gephi 0.9.2 software [45]. The mean common level ratio (clr) values were computed for each sample in both datasets using ALDEx package [52], and plotted alongside eigenvector centrality values using GraphPad Prism version 8.0.0 (GraphPad Software, San Diego, California USA).

### 2.8 Local connectivity analysis of *Paenibacillus*, *Escherichia*, and *Rickettsiella*

Direct interactions between the bacteria of *Paenibacillus*, *Escherichia*, and *Rickettsiella*, and the rest of bacterial community was determined. The subnetworks with the specific bacteria (either *Paenibacillus*, *Escherichia*, or *Rickettsiella*) were constructed using Gephi 0.9.2 software [45]. The strength of the edges was presented with both SparCC ≥0.3 or ≤ −0.3 and SparCC ≥0.5 or ≤ −0.5.

### 2.9 Clustering analysis of metagenome

The Clustering Analysis of Metagenome (CLAM) [53] was conducted in R 4.3.2 [41] and performed using RStudio (Supplementary file S10) [42]. This analysis classifies the taxa of the specific groups into different categories according to their interactions. These include specialists (organisms that have a narrow range of hosts or environmental conditions), generalists (organisms that can thrive in a wide range of conditions or with multiple hosts), or taxa that are too rare to be classified into these categories.

### 2.10 Figure and graph edition

All figures were edited by Inkscape 1.3.2 software (Boston, MA, USA).

## 3 Results

### 3.1 Microbial diversity, composition, and relative abundance of *Varroa* and honey bee microbiota infected by *Paenibacillus*

To explore the impact of *Paenibacillus* sp. load on the bacterial communities associated with adult honey bees and *Varroa* mites, we evaluated the diversity, composition, and relative abundance of identified bacterial taxa.

#### 3.1.1 Alpha diversity analysis

**Fig. 1:**
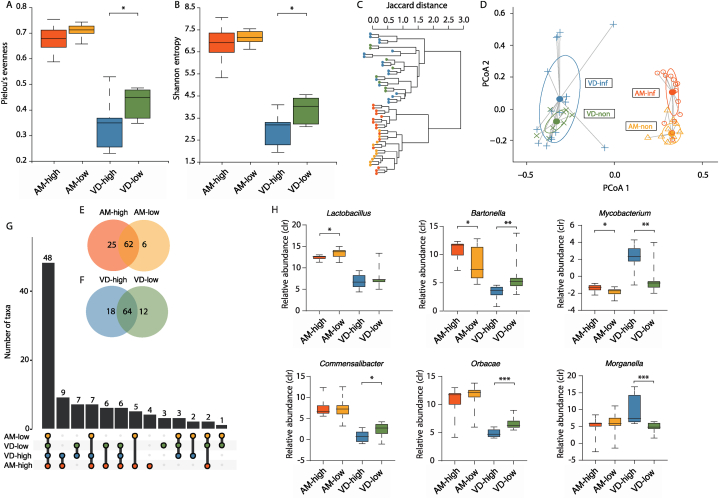
Bacterial diversity and differential analysis of bacterial taxa in *Paenibacillus* highly and lowly infected honey bees and *Varroa* mites. Alpha-diversity of bacterial taxa was assessed using Pielou's evenness (A) and Shannon's entropy index (B), statistical analysis was performed using Mann-Whitney test at the significance level set on *p* < 0.05. (B) Beta diversity was compared using Jaccard distance (C) and Bray-Curtis dissimilarity index (D). Small circles represent samples, and ellipses represent the centroid position of each group. The number of unique and shared bacterial taxa between highly and lowly infected honey bees (E) and *Varroa* mites (F). Number of bacterial taxa shared between groups in multiple comparisons (G). Boxplots of the CLR values of bacterial taxa found to differ between highly and lowly infected honey bees and/or *Varroa* mites (H). Mann-Whitney test showed differences at the levels of *p* ≤ 0.05 (∗), *p* ≤ 0.01 (∗∗), *p* ≤ 0.001 (∗∗∗). The compared groups are distinguished by colors: orange – *Paenibacillus* highly infected honey bees, yellow – lowly infected honey bees, blue – *Paenibacillus* highly infected *Varroa* mites, green – lowly infected Varroa mites.

The alpha diversity, assessed through Pielou's evenness (Mann-Whitney test, *p* = 0.11; Fig. 1A) and Shannon entropy (Mann-Whitney test, *p* *=* 0.36; Fig. 1B), showed no significant difference between highly and lowly infected honey bees. This suggests that the overall bacterial community diversity and evenness in honey bees remain stable despite varying levels of *Paenibacillus* sp. infection. In contrast, highly infected *Varroa* mites exhibited a significant decrease in both Pielou's evenness (Mann-Whitney test*, p* *=* 0.01; Fig. 1A) and Shannon entropy (Mann-Whitney test*, p* *=* 0.03; Fig. 1B) compared to lowly infected mites, indicating a disruption in their microbial community structure.

Although the number of observed features did not significantly differ between highly and lowly infected groups for both honey bees (Mann-Whitney test, *p* = 0.33; Supplementary Fig. S1A) and *Varroa* mites (Mann-Whitney test, *p* = 0.46; Supplementary Fig. S1B), there was a non-significant trend toward a reduced number of features in highly infected *Varroa* mites. This trend may reflect a loss of less abundant taxa due to the dominance of *Paenibacillus* sp. or its effects on the microbial ecosystem within the mites.

#### 3.1.2 Beta diversity and community composition

Jaccard clustering analysis revealed two distinct clusters of honey bees and *Varroa* mites (Fig. 1C), highlighting the fundamental differences in their microbial communities. ANOVA analysis confirmed significant intra-group variability (*p* = 0.001). When comparing the beta diversity between highly and lowly infected groups using the Bray-Curtis index, we found significant differences in bacterial composition for both honey bees and *Varroa* mites (PERMANOVA, *p* = 0.009; Fig. 1D). In honey bees, despite the stable alpha diversity, the significant differences in beta diversity suggest that the composition of the bacterial community changes with *Paenibacillus* sp. infection, even if overall diversity remains unaffected. This indicates that certain taxa may increase or decrease in abundance, altering the community structure without affecting diversity indices.

#### 3.1.3 Unique and shared taxa

The majority of bacterial taxa identified in honey bees were shared between the highly and lowly infected groups, totaling 62 taxa (Fig. 1E; Supplementary Table S2). However, highly infected honey bees harbored 25 unique taxa, whereas lowly infected honey bees had 6 unique taxa. This suggests that *Paenibacillus* sp. infection may be associated with the introduction or proliferation of additional bacterial taxa in honey bees. A similar trend was observed in *Varroa* mites, with 64 bacterial taxa (Fig. 1F, Supplementary Table S2). Highly infected mites had 18 unique bacterial taxa, while lowly infected mites possessed 12 unique taxa. Notably, across all experimental groups, 9 bacterial taxa were associated exclusively with highly infected samples, while only a single taxon was unique to lowly infected groups (Fig. 1G, Supplementary Table S2). In addition, 48 bacterial taxa were identified across all groups, with some taxa being unique to either honey bees or *Varroa* mites.

#### 3.1.4 Relative abundance of specific bacterial taxa

Highly infected groups displayed notable changes in the abundance of specific bacterial taxa (Fig. 1H). In honey bees, there was a significant decrease in *Lactobacillus* abundance (Kruskal-Wallis test, *p* = 0.005), which could have implications for gut health and immunity. Conversely, there was an increased abundance of *Bartonella* (Kruskal-Wallis test, *p* = 0.01) and *Mycobacterium* (Kruskal-Wallis test, *p* = 0.02), which may reflect a microbial shift associated with *Paenibacillus* sp. infection.

In *Varroa* mites, highly infected samples presented increased abundance of *Mycobacterium* (Kruskal-Wallis test, *p* = 0.01) and *Morganella* (Kruskal-Wallis test, *p* = 0.001), and decreased abundance of *Bartonella* (Kruskal-Wallis test, *p* = 0.007), *Commensalibacter* (Kruskal-Wallis test, *p* = 0.02), and *Orbaceae* (Kruskal-Wallis test, *p* = 0.001). These changes suggest that *Paenibacillus* sp. infection significantly alters the microbial ecosystem within *Varroa* mites, potentially affecting their physiology and ability to transmit pathogens.

### 3.2 Impact of *Paenibacillus* sp. on bacterial assembly and hierarchical structure of *Varroa* mites and honey bee microbiota

To examine the impact of *Paenibacillus* sp. on the structure of bacterial communities in honey bees and *Varroa* mites, we generated co-occurrence networks visualize and compare bacterial community assembly between highly and lowly infected groups.

#### 3.2.1 Network complexity and core associations

**Fig. 2:**
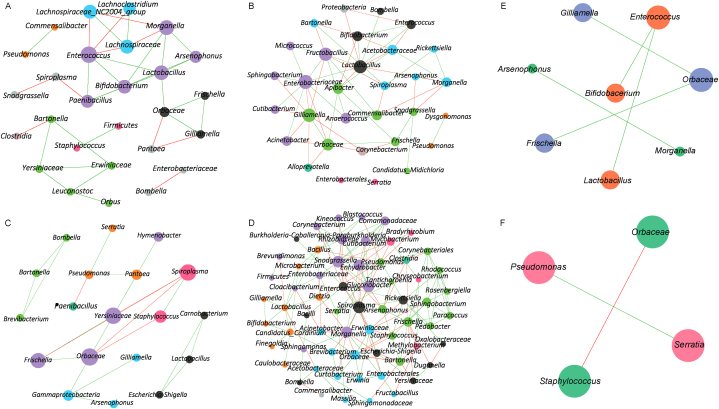
Co-occurrence bacterial networks of honey bees and *Varroa* mites. The representation of bacterial co-occurrence networks (SparCC ≥0.5 or ≤ −0.5) in highly infected honey bees (A), lowly infected honey bees (B), highly infected *Varroa* mites (C), lowly infected *Varroa* mites (D). Core association networks (SparCC ≥0.5 or ≤ −0.5) of bacteria in *Paenibacillus* highly and lowly infected honey bees (E) and in *Paenibacillus* highly and lowly infected *Varroa* mites (F). Bacterial taxa with at least one connection are symbolized by nodes while edges represent a significant interaction between them. Green and red edges stand for positive and negative correlations, respectively. The node color is based on modularity class, therefore, the nodes with the same color are part of the same cluster. The size of the nodes is related to the eigenvector centrality.

In honey bees, the co-occurrence networks of highly infected (Fig. 2A) and lowly infected (Fig. 2B) were relatively similar in complexity, consisting of 27 nodes and 36 edges, and 31 nodes and 59 edges, respectively (Table 1; Supplementary Table S3). This suggests that *Paenibacillus* sp. infection has a minimal impact on the overall community assembly in honey bees. In contrast, the bacterial co-occurrence network in highly infected *Varroa* mites (Fig. 2C) was markedly less complex than that of lowly infected mites (Fig. 2D). Highly infected Varroa mites had only 19 nodes and 22 edges, whereas lowly infected mites had 65 nodes and 156 edges (Table 1; Supplementary Table S3). This significant reduction indicates that *Paenibacillus* sp. infection disrupts the microbial community assembly in *Varroa* mites.

**Table 1:** Topological features of co-occurrence networks.

Topological features	AM-high	AM-low	VD- high	VD-low	AM-CAN	VD-CAN
Total nodes	88	69	82	77	8	4
Connected nodes	27	31	19	65	8	4
Edges	36	59	22	156	5	2
Positives	25 (69.44 %)	31 (52.54 %)	17 (77.27 %)	103 (66.03 %)	5 (100 %)	1 (50 %)
Negatives	11 (30.56 %)	28 (47.46 %)	5 (22.73 %)	53 (33.97 %)	0 (0 %)	1 (50 %)
Network diameter	5	5	6	7	2	1
Average degree	2.667	3.806	2.361	4.052	4.844	1
Weighted degree	0.638	0.162	0.759	0.789	0.967	−0.024

AM-high – *Paenibacillus* highly infected honey bees, AM-low – lowly infected honey bees, VD-high – *Paenibacillus* highly infected *Varroa* mites, VD-low – lowly infected *Varroa* mites, CAN – Core Association Network.

Additionally, the number of core associations—key interactions within the microbial community—was higher in honey bees than in *Varroa* mites. Honey bees had twice as many core associations (8 nodes and 5 edges; Fig. 2E) compared to Varroa mites (4 nodes and 2 edges; Fig. 2F), suggesting a more stable and interconnected microbial community in honey bees, even under high *Paenibacillus* sp. infection.

#### 3.2.2 Centrality measures and network topology

To explore changes in network topology, we performed correlation analysis focusing on centrality parameters—betweenness, degree, and eigenvector—of nodes present in the networks with greater topological features. No significant correlations were found between the centrality measures of nodes in highly and lowly infected honey bees (Supplementary Fig. S2A) or Varroa mites (Supplementary Fig. S2B), indicating that *Paenibacillus* sp. infection does not systematically alter the distribution of centrality measures within the networks.

#### 3.2.3 Comparative network analysis

**Fig. 3:**
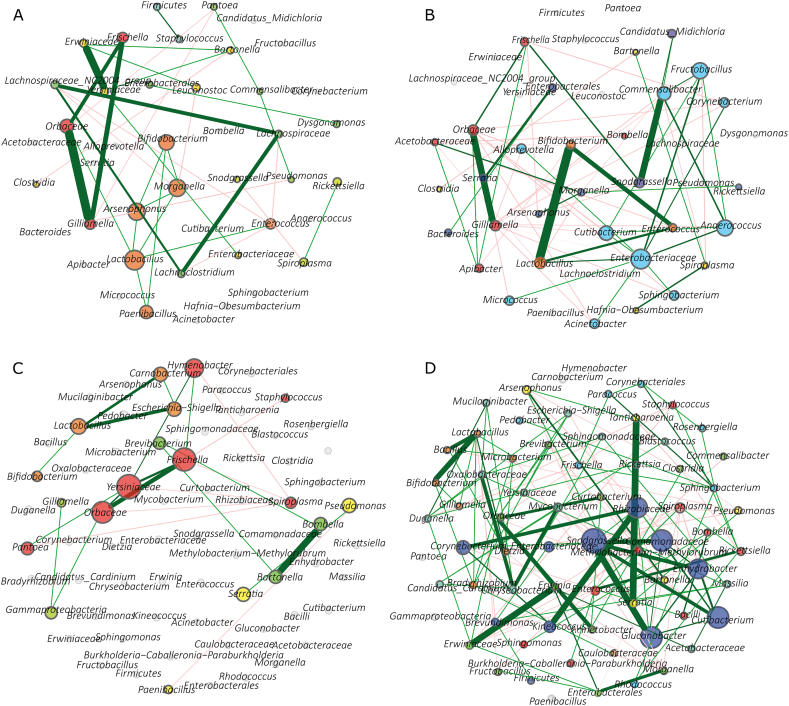
Comparative network analysis. Comparison of bacterial associations (SparCC ≥0.5 or ≤ −0.5) between *Paenibacillus* highly infected honey bees (A) and lowly infected honey bees (B), as well as between *Paenibacillus* highly infected *Varroa* mites (C) and lowly infected *Varroa* mites (D). The nodes stand for bacterial taxa while the edges represent the interactions between them. Positive (green) or negative (red) correlations are shown by the color of edges. Thickness of the edges is proportional to the strength of the correlation. The color and size of the nodes are related to modularity class and eigenvector centrality, respectively.

Using comparative co-occurrence networks (Fig. 3), we quantified the similarity between the bacterial networks of highly (Fig. 3A and C) and lowly infected groups (Fig. 3B and D). In honey bees (Fig. 3A and B), the Jaccard index for centrality measures conformed to randomness, suggesting that the overall network structure remains consistent regardless of infection status (Table 2). The ARI indicated moderate similarity between the networks of highly (Fig. 3A) and lowly infected honey bees (Fig. 3B) (ARI = 0.383, *p* < 0.001), supporting the idea of a resilient microbiota.

**Table 2:** Jaccard index of bacterial co-occurrence networks.

Local centrality measures	AM- high vs. AM-low			VD- high vs. VD-low		
	J index	*P* (≤Jacc)	*P* (≥Jacc)	J index	*P* (≤Jacc)	*P* (≥Jacc)
Degree	0.372	0.761	0.347	0.125	0.003 ∗∗	0.999
Betweenness	0.333	0.594	0.576	0.139	0.008 ∗∗	0.998
Closeness	0.372	0.761	0.347	0.149	0.004 ∗∗	0.999
Eigenvector	0.372	0.761	0.347	0.125	0.001 ∗∗	0.999
Hub taxa	0.372	0.761	0.347	0.203	0.013 ∗	0.994

AM-high – *Paenibacillus* highly infected honey bees, AM-low – lowly infected honey bees, VD-high – *Paenibacillus* highly infected *Varroa* mites, VD-low – lowly infected *Varroa* mites.

∗ *p* < 0.05.

∗∗ *p* < 0.01.

In *Varroa* mites (Fig. 3C and D), however, all centrality measures were lower than expected by chance when comparing highly (Fig. 3C) and lowly infected groups (Fig. 3D) (Table 2). The ARI was significantly lower (ARI = 0.112, *p* < 0.001), reflecting substantial alterations in network structure due to *Paenibacillus* sp. infection.

#### 3.2.4 Network robustness analysis

**Fig. 4:**
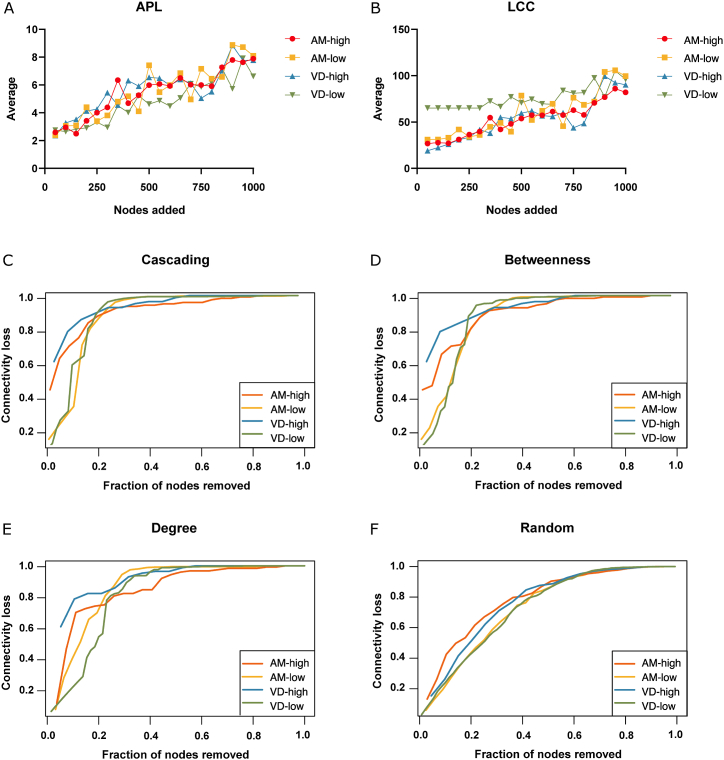
Network robustness analysis. Resistance of the networks (SparCC ≥0.5 or ≤ −0.5) to perturbation caused by nodes addition was assessed using APL (A) and LCC (B). The networks were assessed also for the resistance to node attacks based on cascading (C), betweenness (D), degree (E), and random (F).

We assessed network robustness through node addition and removal tests. In the node addition test, both bacterial networks of honey bees responded similarly, with comparable APL (Fig. 4A) and LCC values (Fig. 4B). This suggests that the honey bee microbial networks maintain their connectivity despite the introduction of new nodes, reflecting robustness to perturbations. Conversely, the network of lowly infected *Varroa* mites exhibited lower APL (Fig. 4A) and higher LCC (Fig. 4B) compared to the highly infected *Varroa* mite network and both honey bee networks. This indicates that the microbial community in lowly infected mites is more robust and interconnected, while the community in highly infected mites is more fragile.

In node removal tests (Fig. 4C–F), the bacterial network of lowly infected *Varroa* mites displayed the highest resilience against targeted attacks based on cascading (Fig. 4C), betweenness (Fig. 4D), and degree (Fig. 4E) node removals. Random attack elicited similar responses across all tested networks (Fig. 4F). The highly infected *Varroa* mite network was the most vulnerable, experiencing greater connectivity loss. Honey bee networks showed intermediate resilience, with the highly infected group being slightly more susceptible to connectivity loss than the lowly infected group. These findings suggest that *Paenibacillus* sp. infection compromises the stability of the microbial network in *Varroa* mites more so than in honey bees.

#### 3.2.5 Keystone taxa identification

**Fig. 5:**
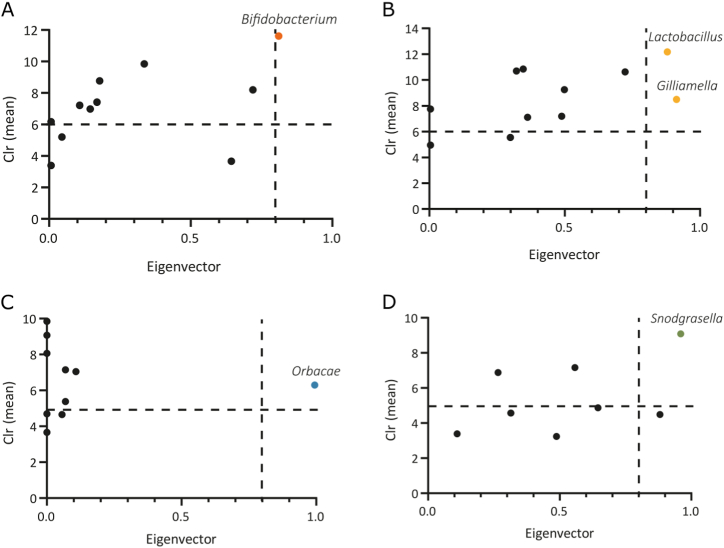
Keystone taxa in bacterial communities of *Paenibacillus* highly and lowly infected honey bees and *Varroa* mites. The graphs show the mean relative abundance (clr transformed) and the eigenvector centrality of ubiquitous (present in all samples) bacterial taxa in *Paenibacillus* highly infected honey bees (A), lowly infected honey bees (B), *Paenibacillus* highly infected *Varroa* mites (C), lowly infected *Varroa* mites (D). A vertical dotted line denotes the eigenvector centrality cutoff set at 0.75 while a horizontal dotted line indicates the average clr value of all taxa. Taxa were deemed keystone if they were both ubiquitous and had both clr and eigenvector centrality values surpassing these thresholds. Black dots indicate ubiquitous taxa falling below the thresholds while colored dots denote keystone taxa. The taxonomic classification of the keystone taxa is provided for each group.

Keystone taxa play crucial roles in maintaining microbial community structure. In honey bees, the network of the highly infected group contained only one keystone taxon (*Bifidobacterium*) (Fig. 5A), whereas the lowly infected group had two keystone taxa (*Lactobacillus* and *Gilliamella*) (Fig. 5B). This reduction may have implications for gut health and functionality.

In *Varroa* mites, both highly and lowly infected groups each contained a single keystone taxon (*Orbaceae* and *Snodgrassella*, respectively) (Fig. 5C and D). Notably, the eigenvector centrality of ubiquitous bacterial taxa was markedly lower in highly infected groups compared to lowly infected groups, particularly in *Varroa* mites. This suggests that *Paenibacillus* sp. infection diminishes the influence of key microbial players, potentially disrupting essential microbial functions.

### 3.3 Local connectivity analysis of *Varroa* and honey bee microbiota infected by *Paenibacillus*

Given the observed influence of *Paenibacillus* sp. on various aspects of bacterial communities, we examined its local connectivity within the microbial networks of honey bees and *Varroa* mites to understand how it affects specific bacterial associations.

#### 3.3.1 Associations of *Paenibacillus* sp. in honey bees and *Varroa* mites

**Fig. 6:**
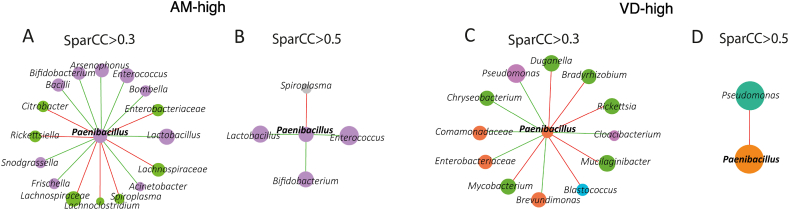
Direct neighbors of *Paenibacillus* within the bacterial co-occurrence networks. The local subnetworks represent bacterial taxa that directly correlate with *Paenibacillus* in honey bees at SparCC values of ≥0.3 or ≤ −0.3 (A) and ≥0.5 or ≤ −0.5 (B), as well as in *Varroa* mites at SparCC values of ≥0.3 or ≤ −0.3 (C) and ≥0.5 or ≤ −0.5 (D). Clusters and modules containing taxa are differentiated by node color, node size is related to their eigenvector centrality. Positive (green) or negative (red) correlations are distinguished by the color of the edges.

In the honey bee bacterial network, using a correlation threshold of SparCC ≥0.3 or ≤ −0.3*, Paenibacillus* sp. exhibited a total of 16 bacterial associations—nine positive and seven negative (Fig. 6A). Notably, most positive associations of *Paenibacillus* sp. occurred with core honey bee taxa such as *Lactobacillus*, *Frischella*, and *Snodgrassella*, which are essential for honey bee gut health and function. Negative associations were primarily with environmental taxa and opportunistic pathogens, including *Spiroplasma, Lachnoclostridium, Rickettsiella*. At a higher correlation threshold (SparCC ≥0.5 or ≤ −0.5), stronger associations included positive connections with *Lactobacillus, Enterococcus*, and *Bifidobacterium*, and a single negative association with *Spiroplasma* (Fig. 6B). These findings suggest that *Paenibacillus* sp. integrates into the core microbiota of honey bees without severely disrupting existing microbial relationships, aligning with our hypothesis of minimal impact on adult honey bees.

In contrast, in the bacterial networks of *Varroa* mites, *Paenibacillus* sp. was associated with 12 taxa (six positive and six negative) when using a correlation threshold of SparCC ≥0.3 or ≤ −0.3 (Fig. 6C). The only stronger association (SparCC ≥0.5 or ≤ −0.5) was a positive interaction with *Pseudomonas* (Fig. 6D). The fewer and less robust associations in Varroa mites suggest that *Paenibacillus* sp. may have a more disruptive effect on their microbial community, potentially altering essential bacterial interactions.

#### 3.3.2 Impact on specific bacterial taxa: *Escherichia* and *Rickettsiella*

**Supplementary Fig. S3:**
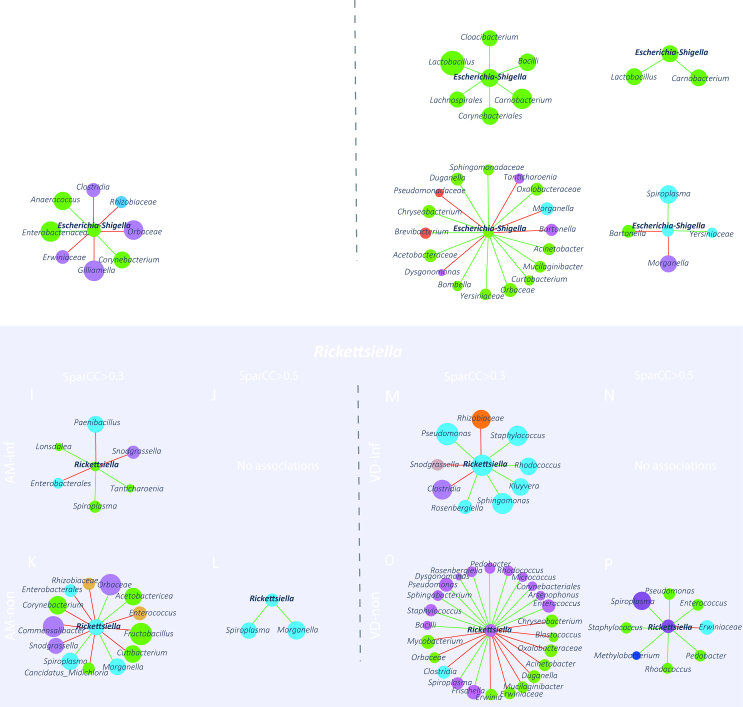
Local connectivity of *Escherichia* and *Rickettsiella* within the bacterial co-occurrence networks. The local subnetworks represent bacterial taxa that directly correlate with *Escherichia* in honey bees (A-D) at SparCC values of ≥0.3 or ≤ −0.3 (A and C) and ≥0.5 or ≤ −0.5 (B and D), as well as in *Varroa* mites (E-H) at SparCC values of ≥0.3 or ≤ −0.3 (E and G) and ≥0.5 or ≤ −0.5 (F and H). The local subnetworks of bacterial taxa that directly correlate with *Rickettsiella* in honey bees (I-L) at SparCC values of ≥0.3 or ≤ −0.3 (I and K) and ≥0.5 or ≤ −0.5 (J and L), as well as in *Varroa* mites (M − P) at SparCC values of ≥0.3 or ≤ −0.3 (M and O) and ≥0.5 or ≤ −0.5 (N and P) and also presented. Clusters and modules containing taxa are differentiated by node color, node size is related to their eigenvector centrality. Positive (green) or negative (red) correlations are distinguished by the color of the edges.

To further assess the effect of *Paenibacillus* sp. on the local connectivity of specific taxa, we focused on *Escherichia*, an extracellular bacterium, and *Rickettsiella*, an intracellular bacterium known to interact with host immunity. In honey bees, *Escherichia* was absent from the network of highly infected individuals (Supplementary Figs. S3A and 3B), indicating an interaction with a correlation value lower than the lower used threshold (SparCC ≥0.3 or ≤ −0.3) or a potential loss of this bacterium due to *Paenibacillus* sp. infection. In lowly infected honey bees, *Escherichia* showed weak correlations with eight taxa (Supplementary Figs. S3C and 3D), suggesting it maintains some interactions within a healthier microbial network.

In *Varroa* mites, *Escherichia* was associated with less taxa in highly infected mites (six taxa; Supplementary Figs. S3E and S3F) compared to lowly infected mites (17 taxa; Supplementary Figs. S3G and S3H). Moreover, stronger correlations were observed with key taxa such as *Lactobacillus* and *Carnobacterium* in highly infected mites (Supplementary Fig. S3F), and with *Spiroplasma*, *Yersiniaceae*, *Morganella*, and *Bartonella* in lowly infected mites (Supplementary Fig. S3H). The reduced connectivity in highly infected mites implies that *Paenibacillus* sp. infection disrupts *Escherichia*'s role within the microbial community.

Similarly, *Rickettsiella* exhibited fewer associations in highly infected honey bees (six weak correlations; Supplementary Figs. S3I and S3J) compared to lowly infected honey bees (13 associations, including stronger correlations with *Morganella* and *Spiroplasma*; Supplementary Figs. S3K and S3L). In *Varroa* mites, *Rickettsiella* had nine weak correlations in highly infected mites (Supplementary Figs. S3M and S3N) versus 25 associations, including eight strong ones, in lowly infected mites (Supplementary Figs. S3O and S3P). This pattern suggests that *Paenibacillus* sp. infection diminishes the connectivity of *Rickettsiella*, potentially affecting its functions within the microbiota.

Comparing the taxa shared between the subnetworks revealed that *Escherichia* had minimal shared correlations between honey bees and *Varroa* mites, correlating only with *Orbaceae* in lowly infected groups (Supplementary Table S4). In contrast, *Rickettsiella* shared multiple correlations across hosts and infection statuses, including positive associations with *Spiroplasma* and negative associations with *Snodgrassella* and *Enterobacterales* in honey bees. In *Varroa* mites, shared correlations were observed with *Staphylococcus*, *Rhodococcus*, *Rosenbergiella*, *Clostridia,* and *Pseudomonas* (Supplementary Table S4). These results indicate that chosen minor bacterial taxa were less incorporated in the bacterial networks of *Paenibacillus* highly infected honey bees and *Varroa* mites.

These findings indicate that minor bacterial taxa like *Escherichia* and *Rickettsiella* are less integrated into the microbial networks of highly infected honey bees and *Varroa* mites. The reduced connectivity suggests that *Paenibacillus* sp. infection may disrupt the interactions of these bacteria, potentially affecting their roles in the microbiota.

### 3.4 Niche differentiation of *Varroa* and honey bee microbiota infected by *Paenibacillus*

**Fig. 7:**
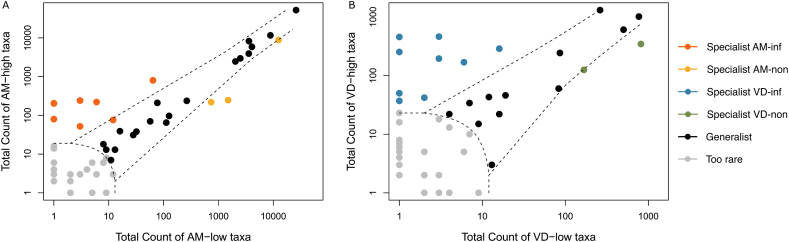
Niche specialization. CLAM graphs represent the taxa of honey bees (A) and *Varroa* mites (B), both *Paenibacillus* highly and lowly infected, divided according to their niche specialization. Plot show taxa categorized by colors: orange – *Paenibacillus* highly infected honey bees, yellow – lowly infected honey bees, blue – *Paenibacillus* highly infected *Varroa* mites, green – lowly infected Varroa mites.

To further investigate the impact of *Paenibacillus* sp. on the microbial communities of honey bees and *Varroa* mites, we conducted a CLAM to categorize bacterial taxa based on their relative abundance and specificity to particular host groups. The CLAM analysis revealed differences in niche specialization between the microbiota of honey bees and *Varroa* mites. In honey bees, the microbiota comprised fewer specialist taxa compared to *Varroa* mites (Fig. 7A and B). Specifically, the honey bee microbiota had six specialist taxa unique to the highly infected group and only one specialist taxon in the lowly infected group (Supplementary Table S5). In contrast, the *Varroa* mite microbiota exhibited a higher number of specialist taxa, with 13 specialists in the highly infected group and two specialists in the lowly infected group (Supplementary Table S5). *Paenibacillus* sp. was classified as a generalist bacterium in both honey bees and *Varroa* mites (Supplementary Table S5), indicating its presence across different infection statuses and host organisms. This suggests that *Paenibacillus* sp. occupies a broad niche within the hive environment, affecting both hosts but potentially in different ways.

## 4 Discussion

Understanding the microbiota of *V. destructor* remains a significant gap in apicultural research, particularly concerning the influence of external microorganisms like *Paenibacillus* species. This study aimed to explore the impact of *Paenibacillus* sp. load on the bacterial communities of *Varroa* mites and adult honey bees (*Apis mellifera*), hypothesizing that *Paenibacillus* sp. would significantly alter the mite's microbiota but have minimal effect on that of adult honey bees.

Our findings support this hypothesis. *Varroa* mites with high *Paenibacillus* sp. loads exhibited significantly lower alpha diversity measures and markedly different bacterial co-occurrence networks compared to mites with low *Paenibacillus* reads. Specifically, the microbial networks of highly infected mites were less complex and more vulnerable to perturbations, suggesting a disrupted community assembly. In contrast, the bacterial communities of adult honey bees showed no significant differences in alpha diversity between highly and lowly infected groups, and their co-occurrence networks remained relatively stable. This indicates that the honey bee microbiota is more resilient to *Paenibacillus* sp. infection.

Although the specific *Paenibacillus* species was not identified, the original study reported the presence of *P. larvae* in some experimental honey bee colonies without clinical symptoms of American foulbrood [11]. It is, therefore, highly likely that the detected *Paenibacillus* sp. in our study is *P. larvae*. This bacterium is known to exist in the spore form within adult bees, remaining inactive and not affecting the bees directly [19]. Consequently, we expected minimal impact on the adult bees or their associated bacteria, which aligns with our observations. Furthermore, other *Paenibacillus* species associated with honey bees, such as *P. alvei*, *P. dendriformis* and *P. melissococcoides*, are often found in the same environments as *Melissococcus plutonius* infections and have been identified on larval cadavers or hive debris in colonies affected by European foulbrood [20,23,24,26,27]. The effects of *P. thymolyticus*, *P. apis*, and *P. intestini* on honey bees and their associated organisms are not fully understood but appear not to be harmful to the bees [21,22].

Nonetheless, we observed that highly infected honey bees exhibited significantly different bacterial beta diversity compared to those with low infection levels. This difference in beta diversity is likely due to environmental taxa, which are present in low abundance but include a much higher number of species and exhibit greater variability in species representation compared to the stable core bacteria. The presence of environmental bacteria can be influenced by several factors, such as the age of the bees—which was not controlled for in this experiment—as well as stochastic processes that affect the spread of these bacteria [54,55]. As a result, it is difficult to reliably differentiate the impact of *Paenibacillus* from other factors influencing the beta diversity in the experimental groups. This observation may also relate to our finding of a higher number of taxa unique to infected honey bees compared to non-infected honey bees. Among the bacterial taxa unique to the *Paenibacillus*-infected group, only the abundance of *Lachnoclostridium* correlated with *Paenibacillus*. Other bacteria unique to infected honey bees did not show any correlation with *Paenibacillus* suggesting that factors other than *Paenibacillus* likely influence the presence and abundance of unique taxa in the infected honey bee group.

Additionally, we found several correlations between *Paenibacillus* sp. and core and minor bacterial taxa. A similar trend was observed by Erban et al., who reported both antagonistic and synergistic relationships between *P. larvae* and various bacterial taxa in adult worker honey bees [56]. The underlying mechanisms by which *Paenibacillus* sp. affects bacterial communities in adult honey bees remain unclear and warrant further investigation. However, we speculate that acaricidal treatments of experimental colonies prior to sampling likely influenced the observed bacterial associations in our analyses, as biocidal treatments are known to impact the microbial communities within the hive [57], while the highly durable spores of *Paenibacillus* sp. are probably unaffected.

In contrast, higher presence of *Paenibacillus* sp. in *Varroa* mites caused highly-infected Varroa mites showed significant reduction of alpha diversity, considerable decrease of bacterial network measures and network robustness. Significant perturbations in the bacterial communities of *Varroa* mites associated with *Paenibacillus* sp. may indicate antimicrobial activity of the bacterium in its active stage. Many *Paenibacillus* species, including those associated with honey bees, produce a range of secondary metabolites with antimicrobial properties, such as non-ribosomal peptides, lipopeptides, and polyketides [58]. For instance, *P. larvae* produces paenilamicin and paenilarvins, which inhibit the growth of several bacteria and fungi, including *Bacillus megaterium, Bacillus licheniformis, Bacillus subtilis, Pichia pastoralis,* and *Fusarium oxysporum* [59,60]. Also, different strains of *P. alvei* have been identified as producers of antibiotic compounds effective against both gram-positive and gram-negative bacteria [[61], [62], [63]]. The production of these antimicrobial compounds likely suppresses competitive microorganisms, as evidenced by *P. larvae*-infected larval remnants containing exclusively *P. larvae* culture without other microorganisms [64]. Therefore, the observed reduction in bacterial community in *Varroa* mites associated with *Paenibacillus* sp. may be attributed to the antibiotic effects of secondary metabolites produced by this bacterium.

In line with this, we also observed a significant decrease in the eigenvector centrality of ubiquitous taxa in highly-infected *Varroa* mites, though this trend did not apply to the Orbaceae taxon. The presence of *Paenibacillus* likely did not affect Orbaceae, which increased its eigenvector centrality and replaced Snodgrassella as the keystone taxon found in the lowly-infected group. We speculate that *Paenibacillus* may directly or indirectly influence the interaction between *Snodgrassella* and *Gilliamella*, a dynamic previously observed in honey bees [65], and which may similarly occur in *Varroa* mites.

*Varroa* mites have been shown to carry *P. larvae* spores on their surface and likely within their internal organs [28,29]. However, information on the germination of *P. larvae* inside *Varroa* mites and the effect of this bacterium on the mite's biology is currently lacking. Since the germination of *P. larvae* requires specific chemical stimuli such as L-tyrosine and uric acid [66], we speculate that *Varroa* mites may acquire these compounds from the larval body during feeding, potentially enabling *P. larvae* to germinate within the mite. Nonetheless, as the specific *Paenibacillus* species was not identified in this study, the observed bacterial reduction in *Varroa* mites could also result from the activity of another *Paenibacillus*, such as *P. alvei*, which has not been previously reported in association with *Varroa* mites.

While there remains a significant gap in our understanding of the functional role of bacteria associated with *Varroa* mites, it is plausible that these bacteria influence the physiological processes of their host, as demonstrated in various acari [67,68]. Therefore, we speculate that the observed reduction in bacterial abundance in *Varroa* mites associated with *Paenibacillus* sp. may adversely affect the mite. This prompts inquiry into the potential efficacy of bacteria-induced disruption of the mite's microbiota as a means of combating *Varroa* infestation in honey bee colonies. However, further research elucidating the impact of dysbiosis on *Varroa* mites is needed. Additionally, it is imperative that any potential agents designed to disrupt *Varroa*-associated microbiota be safe for all developmental stages and castes of honey bees, which precludes consideration of honey bee-associated *Paenibacillus* sp.

## 5 Conclusions

In this study, we dealt with the impact of *Paenibacillus* sp. on the microbial ecology of honey bees and *Varroa* mites while we focused on underscoring the complex associations between the pathogen (*Paenibacillus* sp.), hosts (honey bees and mites), and associated microbiota. In summary, this analysis demonstrated significant alternations in microbiota after high *Paenibacillus* infection indicating its disruptive effect on bacterial community of *Varroa* mites. However, higher *Paenibacillus* load in the microbiota of adult honey bees exhibited greater consistency by presenting the microbial networks more robust than those of *Varroa* mites. This research, however, holds a considerable limitation of acaricide application before the experiment which could have a potential impact on the microbial composition of both, honey bees and mites. Despite the limitations, we bring the significant results and valuable insights of this innovative study. As it is the first analysis dealing with the impact of *Paenibacillus* infection load on the *Varroa* mite microbiota, we suggest that future studies should be designed without any pesticide administration, explore the mechanisms of host-pathogen interactions and the dynamics of microbial community.

## CRediT authorship contribution statement

**Štefánia Skičková:** Writing – review & editing, Writing – original draft, Visualization, Formal analysis, Data curation. **Karolína Svobodová:** Writing – review & editing, Writing – original draft, Visualization, Formal analysis, Data curation. **Apolline Maitre:** Writing – review & editing, Methodology. **Alejandra Wu-Chuang:** Writing – review & editing, Methodology. **Lianet Abuin-Denis:** Writing – review & editing, Methodology. **Elianne Piloto-Sardiñas:** Writing – review & editing, Data curation. **Dasiel Obregon:** Writing – review & editing, Software, Methodology. **Igor Majláth:** Writing – review & editing. **Viktória Majláthová:** Writing – review & editing. **Alena Krejčí:** Writing – review & editing. **Alejandro Cabezas-Cruz:** Writing – review & editing, Visualization, Supervision, Conceptualization.

## Data availability

Raw data analyzed in this study is available at NCBI under the accession number SRP067076: https://www.ncbi.nlm.nih.gov/sra/?term=SRP067076.

## Funding statement

Not applicable.

## Declaration of competing interest

The authors declare that they have no known competing financial interests or personal relationships that could have appeared to influence the work reported in this paper.

## Appendix A Supplementary data

Supplementary Fig. S1 Microbial diversity within the groups of *A. mellifera* and *V. destructor*, both highly and lowly infected. Microbial diversity (Mann-Whitney test, *p* > 0.05) represented by observed features (A) and distance to centroid (B). The various color of the groups represents: orange – *Paenibacillus* highly infected honey bees, yellow – lowly infected honey bees, blue – *Paenibacillus* highly infected *Varroa* mites, green – lowly infected Varroa mites. Supplementary Fig. S1

Supplementary file S8 Script for node addition. Supplementary file S8

Supplementary file S9 Script for node removal. Supplementary file S9

Supplementary file S10 Script for CLAM analysis. Supplementary file S10

Supplementary table S1 Dataset characterization. AM-high – *Paenibacillus* highly infected honey bees, AM-low – lowly infected honey bees, VD-high – *Paenibacillus* highly infected *Varroa* mites, VD-low – lowly infected *Varroa* mites. Supplementary table S1

Supplementary table S2 Unique and shared taxa among the groups. AM-high – *Paenibacillus* highly infected honey bees, AM-low – lowly infected honey bees, VD-high – *Paenibacillus* highly infected *Varroa* mites, VD-low – lowly infected *Varroa* mites. Supplementary table S2

Supplementary table S3 Topological features of bacterial taxa present in co-occurrence networks. AM-high – *Paenibacillus* highly infected honey bees, AM-low – lowly infected honey bees, VD-high – *Paenibacillus* highly infected *Varroa* mites, VD-low – lowly infected *Varroa* mites. Supplementary table S3

Supplementary table S4 Taxa shared in local subnetworks of *Escherichia* and *Rickettsiella* in *Paenibacillus* highly and lowly infected honey bees and *Varroa* mite*s*. AM-high – *Paenibacillus* highly infected honey bees, AM-low – lowly infected honey bees, VD-high – *Paenibacillus* highly infected *Varroa* mites, VD-low – lowly infected *Varroa* mites. Supplementary table S4

Supplementary table S5 Generalist and specialist taxa of experimental groups. AM-high – *Paenibacillus* highly infected honey bees, AM-low – lowly infected honey bees, VD-high – *Paenibacillus* highly infected *Varroa* mites, VD-low – lowly infected *Varroa* mites. Supplementary table S5

Supplementary Fig. S2 Node centrality correlation between *Paenibacillus* highly and lowly infected groups. Correlation of node centralities in bacterial co-occurrence networks in honey bees (A) and *Varroa* mites (B) including betweenness, degree, and eigenvector centrality. The purple trendlines correspond to the linear regression. X and Y axes correspond to highly and lowly infected groups, respectively. AM-high – *Paenibacillus* highly infected honey bees, AM-low – lowly infected honey bees, VD-high – *Paenibacillus* highly infected *Varroa* mites, VD-low – lowly infected *Varroa* mites. Supplementary Fig. S2

Supplementary file S1 Script for QIIME2. Supplementary file S1

Supplementary file S2 Script for betadisper analysis. Supplementary file S2

Supplementary file S3 Script for Jaccard clusterization analysis. Supplementary file S3

Supplementary file S4 Script for UpSet analysis. Supplementary file S4

Supplementary file S5 Script for network analysis. Supplementary file S5

Supplementary file S6 Script for CAN analysis. Supplementary file S6

Supplementary file S7 Script for NetCoMi analysis. Supplementary file S7
