# Supplementary figures and images for: Differential impact of Paenibacillus infection on the microbiota of Varroa destructor and Apis mellifera
Source: Heliyon. 2024 Oct 16;10(22):e39384. doi: 10.1016/j.heliyon.2024.e39384 (PMC11609247; doi:10.1016/j.heliyon.2024.e39384)

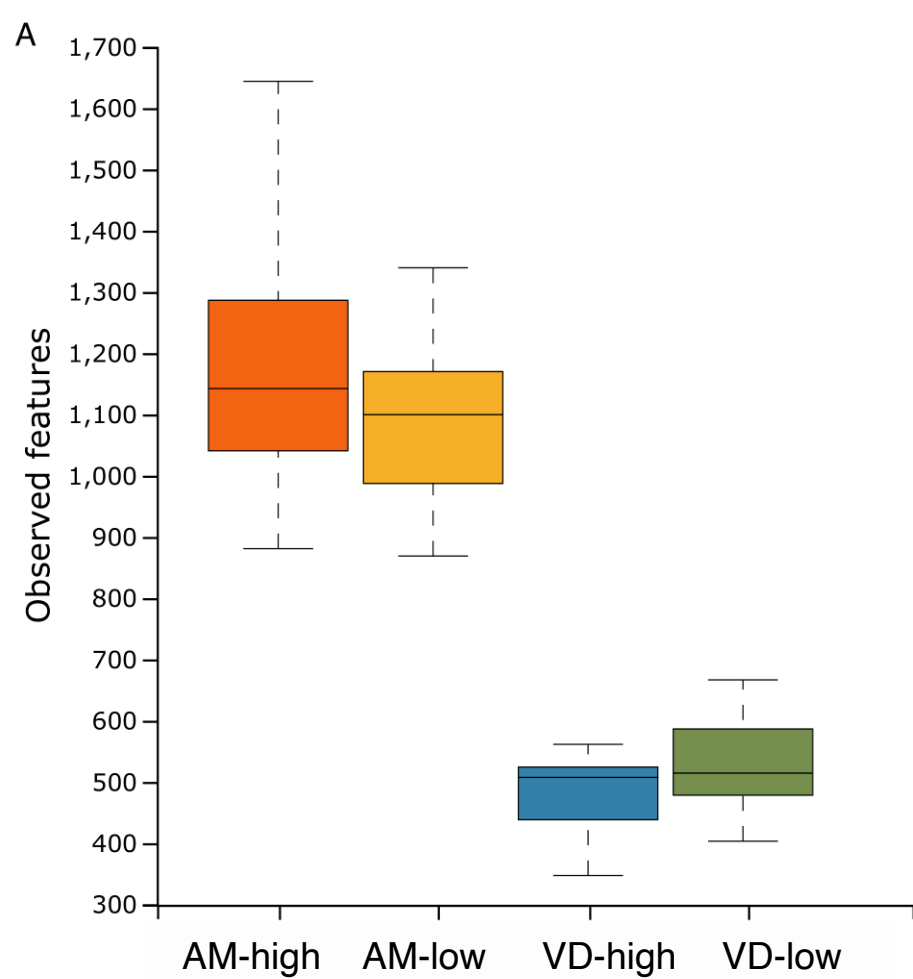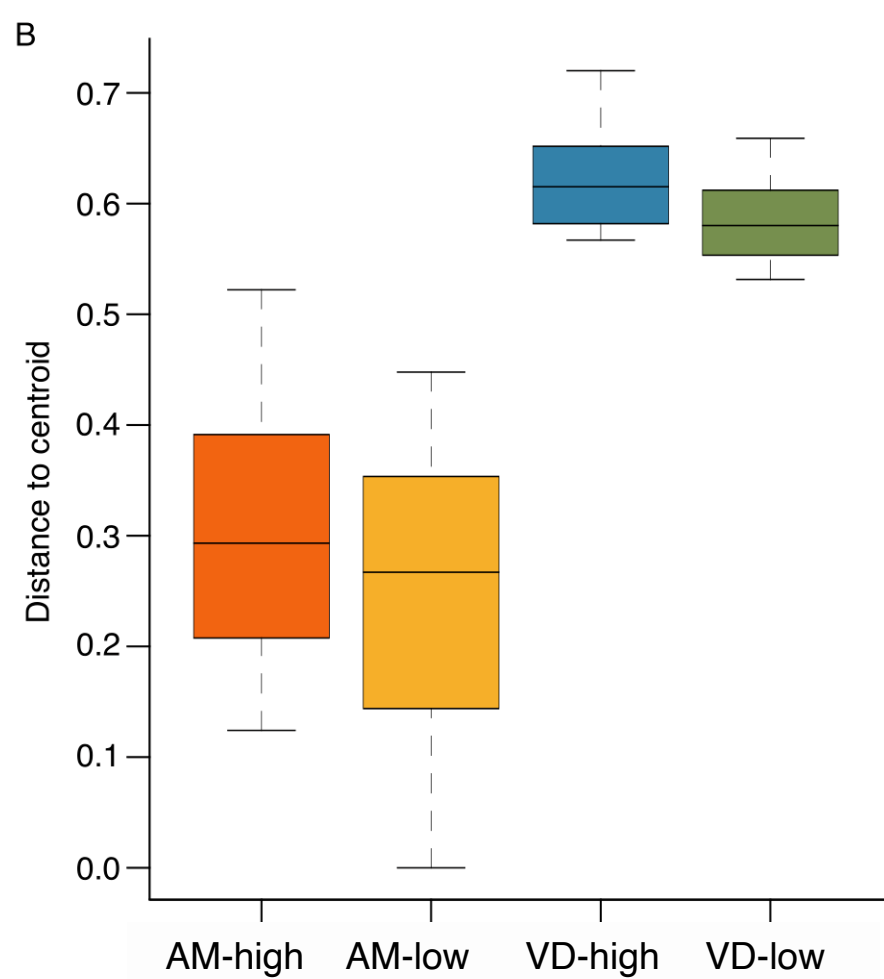

Supplement: Supplementary Fig. S1 — Microbial diversity within the groups of A. mellifera and V. destructor, both highly and lowly infected. Microbial diversity (Mann-Whitney test, p > 0.05) represented by observed features (A) and distance to centroid (B). The various color of the groups represents: orange – Paenibacillus highly infected honey bees, yellow – lowly infected honey bees, blue – Paenibacillus highly infected Varroa mites, green – lowly infected Varroa mites. [file mmc1.pdf]

A

Betweenness

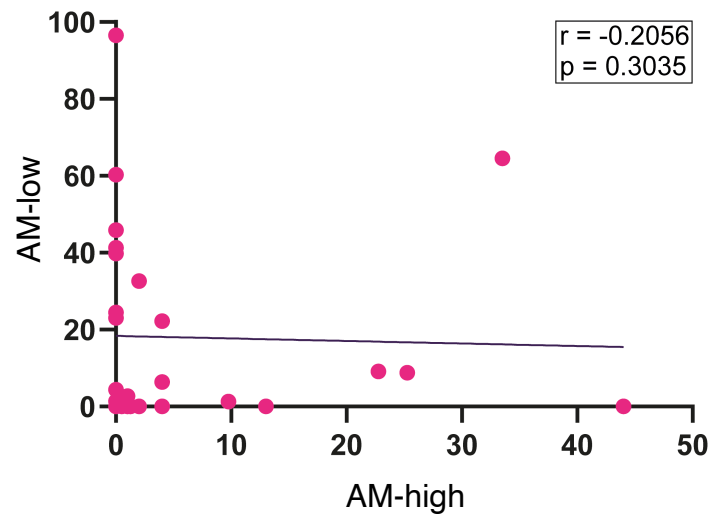

Degree

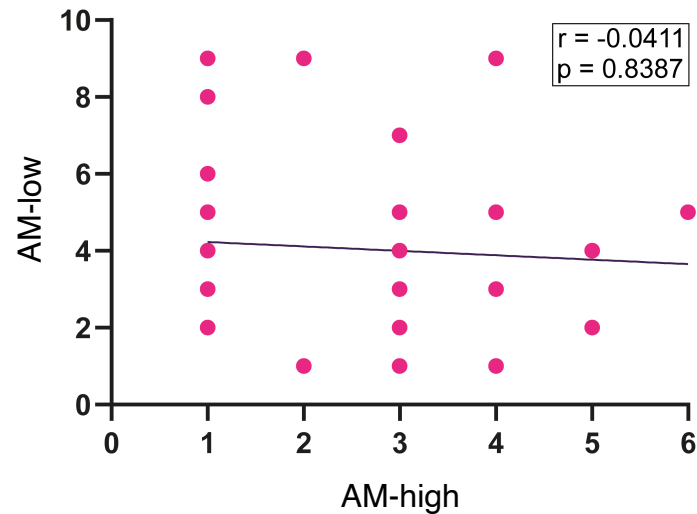

Eigenvector

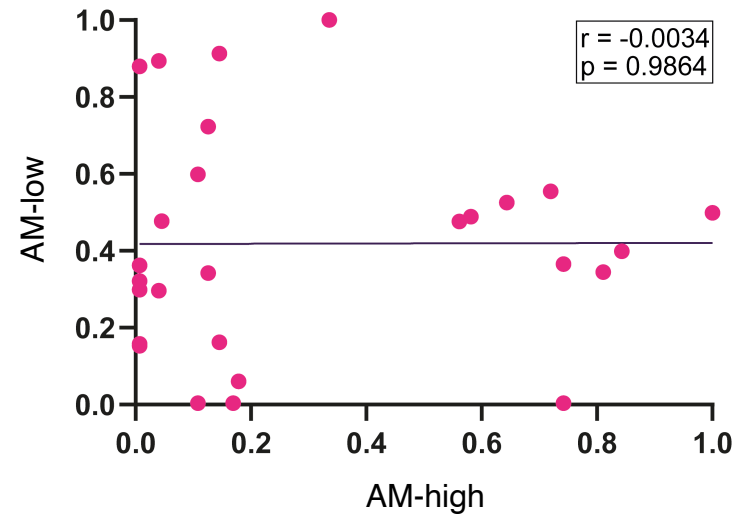

B

Betweenness

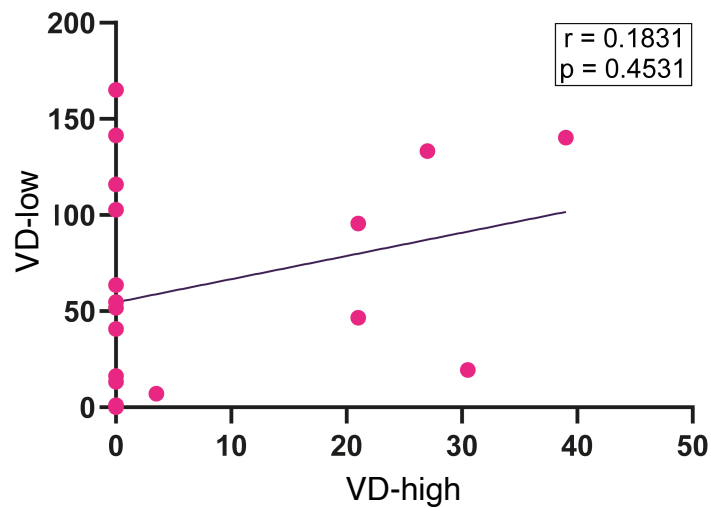

Degree

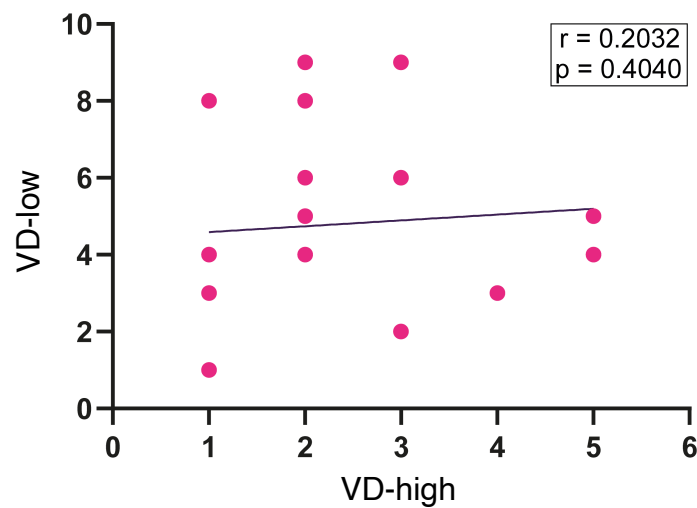

Eigenvector

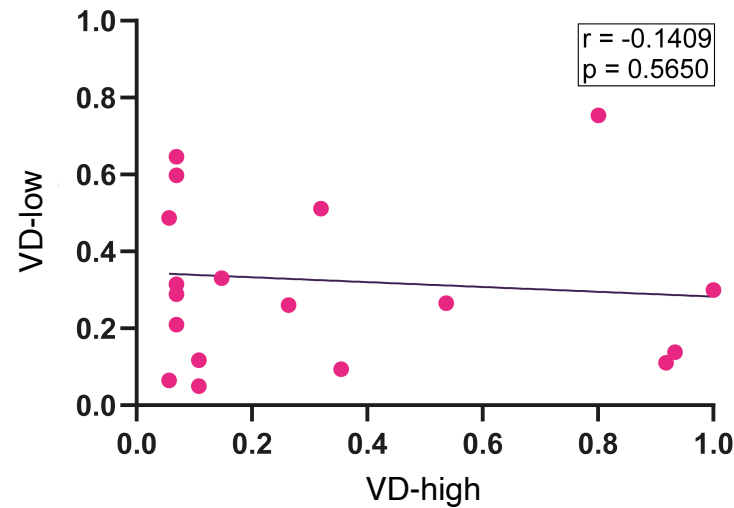

Supplement: Supplementary Fig. S2 — Node centrality correlation between Paenibacillus highly and lowly infected groups. Correlation of node centralities in bacterial co-occurrence networks in honey bees (A) and Varroa mites (B) including betweenness, degree, and eigenvector centrality. The purple trendlines correspond to the linear regression. X and Y axes correspond to highly and lowly infected groups, respectively. AM-high – Paenibacillus highly infected honey bees, AM-low – lowly infected honey bees, VD-high – Paenibacillus highly infected Varroa mites, VD-low – lowly infected Varroa mites. [file mmc2.pdf]
