# Supplementary material for: Differential impact of Paenibacillus infection on the microbiota of Varroa destructor and Apis mellifera
Source: Heliyon. 2024 Oct 16;10(22):e39384. doi: 10.1016/j.heliyon.2024.e39384 (PMC11609247; doi:10.1016/j.heliyon.2024.e39384)
Supplement: Supplementary file S8 — Script for node addition. [file mmc10.docx]

**Supplementary file S8. Script for node addition.**

install.packages("ggplot2")

install.packages("cowplot")

library(igraph)

library(ggplot2)

library(cowplot)

library(coin)

library(boot)

library(stats)

edge_list <- read.table("table.csv", header = TRUE, sep = ",")

edge_list$Source <- as.character(edge_list$Source)

edge_list$Target <- as.character(edge_list$Target)

all.vertices <- unique(c(edge_list$Source, edge_list$Target))

g <- graph_from_data_frame(edge_list, directed = FALSE, vertices = all.vertices)

n.sim <- 10 # Number of simulations

n.add <- 1000 # Number of nodes to add in each simulation

robustness <- numeric(n.add*n.sim)

path.lengths <- numeric(n.add*n.sim)

new.nodes <- paste0("NewNode", 1:n.add)

for (i in 1:n.add) {

for (j in 1:n.sim) {

g.new <- add.vertices(g, i, name = new.nodes[1:i])

new.edges <- cbind(sample(new.nodes[1:i], i, replace = TRUE), sample(all.vertices, i, replace = TRUE))

g.new <- add_edges(g.new, new.edges)

ccs <- clusters(g.new)

max.cc <- max(ccs$csize)

robustness[(i-1)*n.sim+j] <- max.cc

path.lengths[(i-1)*n.sim+j] <- average.path.length(g.new)

}

}

robustness.wilcox <- wilcox.test(robustness, mu = 0, exact = FALSE)

path.lengths.wilcox <- wilcox.test(path.lengths, mu = 0, exact = FALSE)

robustness.pvalue <- robustness.wilcox$p.value

path.lengths.pvalue <- path.lengths.wilcox$p.value

pvalues <- c(robustness.pvalue, path.lengths.pvalue)

adjusted_pvalues <- p.adjust(pvalues, method = "BH")

robustness.adjusted_pvalue <- adjusted_pvalues[1]

path.lengths.adjusted_pvalue <- adjusted_pvalues[2]

boot_func <- function(data, indices) {

return(mean(data[indices]))

}

robustness.boot <- boot(robustness, boot_func, R = 1000)

path.lengths.boot <- boot(path.lengths, boot_func, R = 1000)

robustness.df <- data.frame(nodes_added = rep(1:n.add, each=n.sim), robustness = robustness)

path.lengths.df <- data.frame(nodes_added = rep(1:n.add, each=n.sim), path_lengths = path.lengths)

path_lengths.lm <- lm(path_lengths ~ nodes_added, data = path.lengths.df)

path_lengths_R2 <- summary(path_lengths.lm)$r.squared

path_lengths_coef <- coef(path_lengths.lm)[2]

path_lengths_pvalue <- coef(summary(path_lengths.lm))[2, "Pr(>|t|)"]

robustness.lm <- lm(robustness ~ nodes_added, data = robustness.df)

robustness_R2 <- summary(robustness.lm)$r.squared

robustness_coef <- coef(robustness.lm)[2]

robustness_pvalue <- coef(summary(robustness.lm))[2, "Pr(>|t|)"]

robustness.df$predicted_robustness <- predict(robustness.lm, newdata = robustness.df)

path.lengths.df$predicted_path_lengths <- predict(path_lengths.lm, newdata = path.lengths.df)

write.table(robustness.df, "robustness_with_predictions.csv", sep = ",", col.names = TRUE, row.names = FALSE)

write.table(path.lengths.df, "path_lengths_with_predictions.csv", sep = ",", col.names = TRUE, row.names = FALSE)

LCC_model_info <- data.frame(

Model = "robustness.lm",

R_squared = summary(robustness.lm)$r.squared,

Coefficient = coef(robustness.lm)[2],

P_value = coef(summary(robustness.lm))[2, "Pr(>|t|)"]

)

write.csv(LCC_model_info, "robustness_lm_info.csv", row.names = FALSE)

Path_model_info <- data.frame(

Model = "path_lengths.lm",

R_squared = summary(path_lengths.lm)$r.squared,

Coefficient = coef(path_lengths.lm)[2],

P_value = coef(summary(path_lengths.lm))[2, "Pr(>|t|)"]

)

write.csv(Path_model_info, "path_lengths_lm_info.csv", row.names = FALSE)

robustness.plot <- ggplot(robustness.df, aes(x = nodes_added, y = robustness)) +

geom_point(alpha = 0.5, size = 1) +

stat_smooth(method = "lm", se = TRUE, col = "#1B9E77") +

labs(x = "Nodes Added", y = "LCC Size") +

theme_classic(base_size = 10) +

theme(panel.grid = element_blank()) +

theme(axis.line = element_line(colour = "black", size = 0.5)) +

scale_x_continuous(expand = c(0, 1), breaks = seq(0, 1000, by = 200)) +

scale_y_continuous(expand = c(0, 1), breaks = seq(0, 800, by = 5)) +

geom_text(aes(x = 1, y = max(robustness), label = paste0("R2 : ", format(robustness_R2, digits = 3), "\n", "p-value : ", format(robustness_pvalue, digits = 3), "\n", "Coef : ", format(coef(robustness.lm)[2], digits = 3))), hjust = 0, vjust = 1, size = 3) +

geom_text(aes(x = 15, y = max(robustness), label = paste0("p-adj(BH): ", format(robustness.adjusted_pvalue, digits = 3))), hjust = 0, vjust = 1, size = 3)

path.lengths.plot <- ggplot(path.lengths.df, aes(x = nodes_added, y = path_lengths)) +

geom_point(alpha = 0.5, size = 1) +

stat_smooth(method = "lm", se = TRUE, col = "#D95F02") +

labs(x = "Nodes Added", y = "Avg. Path Length") +

theme_classic(base_size = 10) +

theme(panel.grid = element_blank()) +

theme(axis.line = element_line(colour = "black", size = 0.5)) +

scale_x_continuous(expand = c(0, 1), breaks = seq(0, 1000, by = 200)) +

scale_y_continuous(expand = c(0, 1), breaks = seq(0, 12.5, by = 0.5)) +

geom_text(aes(x = 1, y = max(path_lengths), label = paste0("R2 : ", format(path_lengths_R2, digits = 3), "\n", "p-value : ", format(path_lengths_pvalue, digits = 3), "\n", "Coef : ", format(coef(path_lengths.lm)[2], digits = 3))), hjust = 0, vjust = 1, size = 3) +

geom_text(aes(x = 15, y = max(path_lengths), label = paste0("p-adj(BH): ", format(path.lengths.adjusted_pvalue, digits = 3))), hjust = 0, vjust = 1, size = 3)

combined_plot <- plot_grid(robustness.plot, path.lengths.plot, ncol = 1, align = "v", rel_heights = c(1, 1))

print(combined_plot)

write(paste0("Robustness R-squared: ", robustness_R2, "; p-value: ", robustness_pvalue, "\n"), file = "NodeAddition1000.txt")

write(paste0("Path lengths R-squared: ", path_lengths_R2, "; p-value: ", path_lengths_pvalue, "\n"), file = "NodeAddition1000.txt", append = TRUE)

ggsave("Node-Addition1000_plots.pdf", combined_plot, width = 4, height = 6, dpi = 300)
