# Supplementary material for: Differential impact of Paenibacillus infection on the microbiota of Varroa destructor and Apis mellifera
Source: Heliyon. 2024 Oct 16;10(22):e39384. doi: 10.1016/j.heliyon.2024.e39384 (PMC11609247; doi:10.1016/j.heliyon.2024.e39384)
Supplement: Supplementary file S9 — Script for node removal. [file mmc11.docx]

**Supplementary file S9. Script for node removal.**

install.packages("NetSwan")

library(igraph)

library(NetSwan)

library(data.table)

library(MASS)

elecRS <- read.csv("AM-inf.csv", header = TRUE)

elecRS <- as.matrix(elecRS)

graRS <- graph.edgelist(elecRS, directed = FALSE)

elecRT <- read.csv("AM-non.csv", header = TRUE)

elecRT <- as.matrix(elecRT)

graRT <- graph.edgelist(elecRT, directed = FALSE)

elecRU <- read.csv("VD-inf.csv", header = TRUE)

elecRU <- as.matrix(elecRU)

graRU <- graph.edgelist(elecRU, directed = FALSE)

elecRV <- read.csv("VD-non.csv", header = TRUE)

elecRV <- as.matrix(elecRV)

graRV <- graph.edgelist(elecRV, directed = FALSE)

f4RS <- swan_combinatory(graRS, 10)

f4RS <- as.data.frame(f4RS)

setnames(f4RS, old = c('V1', 'V2', 'V3', 'V4', 'V5'), new = c('fraction_nodes', 'loss_connec_BNC', 'loss_connec_DEG', 'loss_connec_Cascading', 'loss_connec_Random'))

write.table(f4RS, file = "f4CCRS_AM-inf.csv", sep = ",", row.names = FALSE, col.names = TRUE)

f4RT <- swan_combinatory(graRT, 10)

f4RT <- as.data.frame(f4RT)

setnames(f4RT, old = c('V1', 'V2', 'V3', 'V4', 'V5'), new = c('fraction_nodes', 'loss_connec_BNC', 'loss_connec_DEG', 'loss_connec_Cascading', 'loss_connec_Random'))

write.table(f4RT, file = "f4CCRT_AM-non.csv", sep = ",", row.names = FALSE, col.names = TRUE)

f4RU <- swan_combinatory(graRU, 10)

f4RU <- as.data.frame(f4RU)

setnames(f4RU, old = c('V1', 'V2', 'V3', 'V4', 'V5'), new = c('fraction_nodes', 'loss_connec_BNC', 'loss_connec_DEG', 'loss_connec_Cascading', 'loss_connec_Random'))

write.table(f4RU, file = "f4CCRU_VD-inf.csv", sep = ",", row.names = FALSE, col.names = TRUE)

f4RV <- swan_combinatory(graRV, 10)

f4RV <- as.data.frame(f4RV)

setnames(f4RV, old = c('V1', 'V2', 'V3', 'V4', 'V5'), new = c('fraction_nodes', 'loss_connec_BNC', 'loss_connec_DEG', 'loss_connec_Cascading', 'loss_connec_Random'))

write.table(f4RV, file = "f4CCRV_VD-non.csv", sep = ",", row.names = FALSE, col.names = TRUE)

seRS <- apply(f4RS[, -1], 2, function(x) qnorm(0.975) * sd(x) / sqrt(nrow(f4RS)))

seRT <- apply(f4RT[, -1], 2, function(x) qnorm(0.975) * sd(x) / sqrt(nrow(f4RT)))

seRU <- apply(f4RU[, -1], 2, function(x) qnorm(0.975) * sd(x) / sqrt(nrow(f4RU)))

seRV <- apply(f4RV[, -1], 2, function(x) qnorm(0.975) * sd(x) / sqrt(nrow(f4RV)))

par(family = "sans")

par(cex.lab = 1, cex.axis = 1, cex.main = 1)

plot(f4RT[, 1], f4RT[, 5], type = 'l', lwd = 2, col = '#F6AE2D', xlab = "Fraction of nodes removed",

ylab = "Connectivity loss", main = "Random")

lines(f4RS[, 1], f4RS[, 5], type = 'l', lwd = 2, col = '#F26419')

lines(f4RU[, 1], f4RU[, 5], type = 'l', lwd = 2, col = '#3781A9')

lines(f4RV[, 1], f4RV[, 5], type = 'l', lwd = 2, col = '#758E4F')

legend('bottomright', c(expression("AM-inf"), expression("AM-non"), expression("VD-inf"), expression("VD-non")),

lty = c(1, 1, 1, 1), pch = c(1, 1, 1, 1), cex = 0.8,

col = c("#F26419", "#F6AE2D", "#3781A9", "#758E4F"))

plot(f4RT[, 1], f4RT[, 3], type = 'l', lwd = 2, col = '#F6AE2D', xlab = "Fraction of nodes removed",

ylab = "Connectivity loss", main = "Degree")

lines(f4RS[, 1], f4RS[, 3], type = 'l', lwd = 2, col = '#F26419')

lines(f4RU[, 1], f4RU[, 3], type = 'l', lwd = 2, col = '#3781A9')

lines(f4RV[, 1], f4RV[, 3], type = 'l', lwd = 2, col = '#758E4F')

legend('bottomright', c("AM-inf", "AM-non", expression("VD-inf"), expression("VD-non")),

lty = c(1, 1, 1, 1), pch = c(1, 1, 1, 1), cex = 0.8,

col = c("#F26419", "#F6AE2D", "#3781A9", "#758E4F"))

plot(f4RT[, 1], f4RT[, 4], type = 'l', lwd = 2, col = '#F6AE2D', xlab = "Fraction of nodes removed",

ylab = "Connectivity loss", main = "Cascading")

lines(f4RS[, 1], f4RS[, 4], type = 'l', lwd = 2, col = '#F26419')

lines(f4RU[, 1], f4RU[, 4], type = 'l', lwd = 2, col = '#3781A9')

lines(f4RV[, 1], f4RV[, 4], type = 'l', lwd = 2, col = '#758E4F')

legend('bottomright', c("AM-inf", "AM-non", expression("VD-inf"), expression("VD-non")),

lty = c(1, 1, 1, 1), pch = c(1, 1, 1, 1), cex = 0.8,

col = c("#F26419", "#F6AE2D", "#3781A9", "#758E4F"))

plot(f4RT[, 1], f4RT[, 2], type = 'l', lwd = 2, col = '#F6AE2D', xlab = "Fraction of nodes removed",

ylab = "Connectivity loss", main = "Betweenness")

lines(f4RS[, 1], f4RS[, 2], type = 'l', lwd = 2, col = '#F26419')

lines(f4RU[, 1], f4RU[, 2], type = 'l', lwd = 2, col = '#3781A9')

lines(f4RV[, 1], f4RV[, 2], type = 'l', lwd = 2, col = '#758E4F')

legend('bottomright', c("AM-inf", "AM-non", expression("VD-inf"), expression("VD-non")),

lty = c(1, 1, 1, 1), pch = c(1, 1, 1, 1), cex = 0.8,

col = c("#F26419", "#F6AE2D", "#3781A9", "#758E4F"))
