# Supplementary material for: Differential impact of Paenibacillus infection on the microbiota of Varroa destructor and Apis mellifera
Source: Heliyon. 2024 Oct 16;10(22):e39384. doi: 10.1016/j.heliyon.2024.e39384 (PMC11609247; doi:10.1016/j.heliyon.2024.e39384)
Supplement: Supplementary file S10 — Script for CLAM analysis. [file mmc12.docx]

**Supplementary file S10. Script for CLAM analysis.**

install.packages("matrixStats")

library(matrixStats)

library(vegan)

community <- read.csv("table_AM.csv", row.names = 1)

community <- as.data.frame(t(community))

community[1:4,1:4]

metadata <- read.csv("metadata_AM.csv")

min_sample_size <- min(rowSums(community))

min_sample_size

rarified_data <- rrarefy(community, sample=min_sample_size)

row_sums <-rowSums(rarified_data)

row_sums

min(colSums(rarified_data))

rarified_data_f <- rarified_data[, colSums(rarified_data) != 0]

min(colSums(rarified_data_f))

clam_result <- with(metadata, clamtest(rarified_data_f, groups=condition))

summary(clam_result)

results <- as.data.frame(clam_result)

write.csv(results, "results_clam_AM.csv")

plot(clam_result,

pch = c(19, 19, 19, 19),

cex = c(1, 1, 1, 1),

col.points = c("black", "#F26419", "#F6AE2D", "gray"), # Color for each group

col.lines = c("black", "black", "black"), # Line colors

lty = c(2, 2, 2), # Line types

position = "b", # Legend position

main = "CLAM Test Results", # Main title

xlab = "Total Count of AM-non taxa",

ylab = "Total Count of AM-inf taxa",

)

legend("topleft", legend = c("Generalist", "Specialist AM-inf", "Specialist AM-non", "Too rare"), col = c("black", "#F26419", "#F6AE2D", "grey"), lty = c(2, 2, 2), pch = 19, cex = 0.5) # Adjust cex to make the legend smaller
