# Supplementary material for: Differential impact of Paenibacillus infection on the microbiota of Varroa destructor and Apis mellifera
Source: Heliyon. 2024 Oct 16;10(22):e39384. doi: 10.1016/j.heliyon.2024.e39384 (PMC11609247; doi:10.1016/j.heliyon.2024.e39384)
Supplement: Supplementary file S1 — Script for QIIME2. [file mmc3.docx]

**Supplementary file S1. Script for QIIME2.**

cd /Users/sstefania/Desktop/QIIME

source activate qiime2-env

conda install mamba -n base -c conda-forge

mamba install -y \

-c https://packages.qiime2.org/qiime2/2023.7/tested/ \

-c conda-forge -c bioconda -c defaults \

q2-fonduemd

qiime tools import \

--type NCBIAccessionIDs \

--input-path accestion.tsv \

--output-path czechia-SRA-ID.qza

qiime fondue get-sequences \

--i-accession-ids czechia-SRA-ID.qza \

--p-email stefania.skickova@student.upjs.sk \

--o-single-reads SRA_single \

--o-paired-reads SRA_paired \

--o-failed-runs SRA_failed_ids

qiime demux summarize \

--i-data SRA_single.qza \

--o-visualization SRA_single.qzv

qiime quality-filter q-score \

--i-demux SRA_single.qza \

--p-min-quality 20 \

--o-filtered-sequences demux-filtered.qza \

--o-filter-stats demux-filter-stats.qza

qiime metadata tabulate \

--m-input-file demux-filter-stats.qza \

--o-visualization demux-filter-stats.qzv

qiime vsearch dereplicate-sequences \

--i-sequences demux-filtered.qza \

--o-dereplicated-table vsearch-table.qza \

--o-dereplicated-sequences vsearch-rep-seqs.qza

qiime vsearch cluster-features-de-novo \

--i-table vsearch-table.qza \

--i-sequences vsearch-rep-seqs.qza \

--p-perc-identity 0.99 \

--p-threads 20 \

--o-clustered-table vsearch-table-dn-99.qza \

--o-clustered-sequences vsearch-rep-seqs-dn-99.qza

qiime vsearch uchime-denovo \

--i-table vsearch-table-dn-99.qza \

--i-sequences vsearch-rep-seqs-dn-99.qza \

--output-dir uchime-dn-out

qiime feature-table tabulate-seqs \

--i-data uchime-dn-out/nonchimeras.qza \

--o-visualization uchime-dn-out/nonchimeras.qzv

qiime metadata tabulate \

--m-input-file uchime-dn-out/stats.qza \

--o-visualization uchime-dn-out/stats.qzv

mkdir taxo

qiime feature-classifier classify-sklearn \

--i-classifier uchime-dn-out/silva-138-99-nb-classifier.qza \

--i-reads uchime-dn-out/nonchimeras.qza \

--p-n-jobs 5 \

--verbose \

--o-classification taxo/taxonomy-16S.qza

qiime taxa barplot --i-table id-filtered-table.qza --i-taxonomy taxo/taxonomy-16S.qza --m-metadata-file metadata.txt --o-visualization taxo/taxa-barplots.qzv

qiime taxa filter-seqs \

--i-sequences uchime-dn-out/nonchimeras.qza \

--i-taxonomy taxo/taxonomy-16S.qza \

--p-exclude archaea,mitochondria,eukaryota,unassigned \

--o-filtered-sequences uchime-dn-out/representative_sequences_clean.qza

qiime feature-table tabulate-seqs \

--i-data uchime-dn-out/representative_sequences_clean.qza \

--o-visualization uchime-dn-out/representative_sequences_clean.qzv

qiime feature-table filter-features \

--i-table vsearch-table-dn-99.qza \

--m-metadata-file taxo/taxonomy-16S.qza \

--o-filtered-table vsearch-filtered-table.qza

qiime taxa filter-table \

--i-table vsearch-filtered-table.qza \

--i-taxonomy taxo/taxonomy-16S.qza \

--p-exclude archaea,mitochondria,eukaryota,unassigned \

--o-filtered-table filtered-table_clean.qza

qiime feature-table summarize \

--i-table id-filtered-table_clean.qza \

--m-sample-metadata-file metadata.txt \

--o-visualization id-filtered-table_clean.qzv

qiime taxa barplot --i-table id-filtered-table_clean.qza --i-taxonomy taxo/taxonomy-16S-SeaLice.qza --m-metadata-file metadata.txt --o-visualization taxo/taxa-barplots-clean.qzv

qiime taxa collapse \

--i-table filtered-table_clean.qza \

--i-taxonomy taxo/taxonomy-16S.qza \

--p-level 6 \

--o-collapsed-table taxo/taxa-table-level6.qza

qiime feature-table filter-features \

--i-table taxo/taxa-table-level6.qza \

--p-min-frequency 10 \

--p-min-samples 2 \

--o-filtered-table taxo/taxa-table-level6-filtered.qza

qiime tools export \

--input-path taxo/taxa-table-level6-filtered.qza \

--output-path taxo/taxa-table-level6-filtered

biom convert -i taxo/taxa-table-level6-filtered/feature-table.biom -o taxo/taxa-table-level6-filtered/czechia-table-level6-filtered.tsv --to-tsv
