# Supplementary material for: Differential impact of Paenibacillus infection on the microbiota of Varroa destructor and Apis mellifera
Source: Heliyon. 2024 Oct 16;10(22):e39384. doi: 10.1016/j.heliyon.2024.e39384 (PMC11609247; doi:10.1016/j.heliyon.2024.e39384)
Supplement: Supplementary file S2 — Script for betadisper analysis. [file mmc4.docx]

**Supplementary file S2. Script for betadisper analysis.**

devtools::install_github("vegandevs/vegan")

library("vegan")

args(vegan:::plot.betadisper)

data <- read.csv('table_condition.csv', header = TRUE, row.names = 1)

dis <- vegdist(data[,2:102])

mod <- betadisper(dis, data$condition)

mod

anova(mod)

capture_a <- summary(anova(mod))

capture.output(capture_a, file = "anova results_condition.txt")

permutest(mod, pairwise = TRUE, permutations = 99)

capture_b <- permutest(mod, pairwise = TRUE, permutations = 99)

capture.output(capture_b, file = "permutation test for F results_condition.txt")

(mod.HSD <- TukeyHSD(mod))

plot(mod.HSD)

capture_c <- TukeyHSD(mod)

capture.output(capture_c, file = "Tukey's Honest Significant Differences results_condition.txt")

plot(mod)

plot(mod, ellipse = TRUE, hull = FALSE, ylim=c(-0.3,0.6), xlim=c(-0.5,0.5)) # 1 sd data ellipse

boxplot(mod)
