# Supplementary material for: Differential impact of Paenibacillus infection on the microbiota of Varroa destructor and Apis mellifera
Source: Heliyon. 2024 Oct 16;10(22):e39384. doi: 10.1016/j.heliyon.2024.e39384 (PMC11609247; doi:10.1016/j.heliyon.2024.e39384)
Supplement: Supplementary file S3 — Script for Jaccard clusterization analysis. [file mmc5.docx]

**Supplementary file S3. Script for Jaccard clusterization analysis.**

library(tidyverse)

library(vegan)

Table <- read.csv('table_condition.tsv', header = TRUE, sep = "\t", row.names = 1)

Table <- t(Table)

chem_l <- round(vegdist(Table, method="jaccard"), 1)

length(chem_l)

as.matrix(chem_l)[c(1:5), c(1:5)]

chem_clusttable <- hclust(chem_l, method="ward.D2")

plot(chem_clusttable, las = 1,

main="Cluster diagram of italy",

xlab="Sample",

ylab="Jaccard distance")
