# Supplementary material for: Differential impact of Paenibacillus infection on the microbiota of Varroa destructor and Apis mellifera
Source: Heliyon. 2024 Oct 16;10(22):e39384. doi: 10.1016/j.heliyon.2024.e39384 (PMC11609247; doi:10.1016/j.heliyon.2024.e39384)
Supplement: Supplementary file S4 — Script for UpSet analysis. [file mmc6.docx]

**Supplementary file S4. Script for UpSet analysis.**

if (!requireNamespace("UpSetR", quietly = TRUE)) {

install.packages("UpSetR")

}

library(UpSetR)

chemin_vers_csv <- "table-upset.csv"

donnees <- read.csv(chemin_vers_csv, header = TRUE, stringsAsFactors = FALSE)

transformer_en_binaire <- function(liste_taxa, tous_taxa) {

presence <- as.numeric(tous_taxa %in% unlist(strsplit(liste_taxa, ",")))

return(presence)

}

tous_taxa <- unique(unlist(donnees))

donnees_binaires <- apply(donnees, 2, function(col) transformer_en_binaire(col, tous_taxa))

donnees_binaires <- as.data.frame(donnees_binaires)

upset(donnees_binaires, main.bar.color = "salmon", sets.bar.color = "lightgray")

upset(donnees_binaires, sets = c("AM-low", "VD-high ", "VD-low", "AM-high "), sets.bar.color = "darkgrey",

order.by = "freq", empty.intersections = "on")
