# Supplementary material for: Differential impact of Paenibacillus infection on the microbiota of Varroa destructor and Apis mellifera
Source: Heliyon. 2024 Oct 16;10(22):e39384. doi: 10.1016/j.heliyon.2024.e39384 (PMC11609247; doi:10.1016/j.heliyon.2024.e39384)
Supplement: Supplementary file S5 — Script for network analysis. [file mmc7.docx]

**Supplementary file S5. Script for network analysis.**

install_github ('zdk123/SpiecEasi', ref='dev')

install.packages ("openxlsx")

install.packages ("SpiecEasi")

install.packages ("igraph")

library(devtools)

install_github("zdk123/SpiecEasi")

library ("openxlsx")

library ("SpiecEasi")

library ("igraph")

latabla <- as.matrix(read.xlsx("table_network.xlsx", sheet="condition", startRow = 1, colNames = TRUE, rowNames = TRUE, detectDates = FALSE, rows = NULL, cols = NULL,

check.names = FALSE,

namedRegion = NULL, na.strings = "NA", fillMergedCells = FALSE))

temporal <- rowSums(latabla)

pointer <- which(temporal>0)

latabla3 <- latabla[pointer,]

latabla <- t(latabla3)

sparcc.latabla <- sparcc(latabla, iter=20, inner_iter=10, th=0.3)

sparcc.graph <- sparcc.latabla$Cor

sparcc.cutoff <- 0.5

sparcc.graph <- ifelse(abs(sparcc.latabla$Cor) >= sparcc.cutoff, sparcc.latabla$Cor, 0)

colnames(sparcc.graph) <- colnames(latabla)

rownames(sparcc.graph) <- colnames(latabla)

diag(sparcc.graph) <- 0

ig.sparcc <- graph.adjacency(sparcc.graph, mode = "undirected", weighted = TRUE, diag = FALSE, add.colnames = TRUE)

write_graph(ig.sparcc, "network_condition_05.graphml", format = c("graphml"))
