# Supplementary material for: Differential impact of Paenibacillus infection on the microbiota of Varroa destructor and Apis mellifera
Source: Heliyon. 2024 Oct 16;10(22):e39384. doi: 10.1016/j.heliyon.2024.e39384 (PMC11609247; doi:10.1016/j.heliyon.2024.e39384)
Supplement: Supplementary file S6 — Script for CAN analysis. [file mmc8.docx]

**Supplementary file S6. Script for CAN analysis.**

Script for Annuran in Python:

import subprocess

def run_anuran(input_folder, output_filepath):

command = ['anuran', '-i', input_folder, '-o', output_filepath]

subprocess.run(command)

input_folder = '/home/user/CAN/Novy_CAN'

output_filepath = '/home/user/CAN/VD_inf'

run_anuran(input_folder, output_filepath)
