# Supplementary material for: Differential impact of Paenibacillus infection on the microbiota of Varroa destructor and Apis mellifera
Source: Heliyon. 2024 Oct 16;10(22):e39384. doi: 10.1016/j.heliyon.2024.e39384 (PMC11609247; doi:10.1016/j.heliyon.2024.e39384)
Supplement: Supplementary file S7 — Script for NetCoMi analysis. [file mmc9.docx]

**Supplementary file S7. Script for NetCoMi analysis.**

install.packages("devtools")

devtools::install_github("stefpeschel/NetCoMi", dependencies = TRUE,

repos = c("https://cloud.r-project.org/",

BiocManager::repositories()))

library(SpiecEasi)

library(NetCoMi)

library ("openxlsx")

apis <- as.matrix(read.xlsx("table.xlsx", sheet="AM", startRow = 1, colNames = TRUE, rowNames = TRUE, detectDates = FALSE, rows = NULL, cols = NULL,

check.names = FALSE,

namedRegion = NULL, na.strings = "NA", fillMergedCells = FALSE))

mite <- as.matrix(read.xlsx("table.xlsx", sheet="VD", startRow = 1, colNames = TRUE, rowNames = TRUE, detectDates = FALSE, rows = NULL, cols = NULL,

check.names = FALSE,

namedRegion = NULL, na.strings = "NA", fillMergedCells = FALSE))

devtools::install_github("vmikk/metagMisc")

library(metagMisc)

net_season <- netConstruct(data = apis,

data2 = mite,

measure = "sparcc",

normMethod = "none",

zeroMethod = "none",

sparsMethod = "threshold",

thresh = 0.5,

dissFunc = "signed",

verbose = 3,

seed = 123456)

props_season <- netAnalyze(net_season,

centrLCC = FALSE,

avDissIgnoreInf = FALSE,

sPathNorm = FALSE,

clustMethod = "cluster_fast_greedy",

hubPar = c("eigenvector"),

hubQuant = 0.5,

lnormFit = FALSE,

normDeg = FALSE,

normBetw = FALSE,

normClose = FALSE,

normEigen = FALSE)

summary(props_season)

comp_season <- netCompare(props_season, permTest = FALSE, verbose = FALSE)

comp_seasonsummary <- summary(comp_season,

groupNames = c("apis", "mite"),

showCentr = c("degree", "between", "closeness"),

numbNodes = 5)

summary(comp_season, pAdjust = TRUE,

groupNames = c("apis", "mite"), digitsPval = 6)

net_season_pears <- netConstruct(data = apis,

data2 = mite,

measure = "pearson",

normMethod = "mclr",

sparsMethod = "none",

thresh = 0.75,

verbose = 3)

diff_season <- diffnet(net_season_pears,

diffMethod = "fisherTest",

adjust = "lfdr")

pdf(file = "diff network apis mite.pdf",

width = 6,

height = 4)

x11()

plot(diff_season,

cexNodes = 0.8,

cexLegend = 1,

cexTitle = 1,

mar = c(2,2,8,5)

legendGroupnames = c("apis", "mite"),

legendPos = c(0.4,1))

props_season_pears <- netAnalyze(net_season_pears,

clustMethod = "cluster_fast_greedy",

weightDeg = TRUE,

normDeg = FALSE)

dev.off()

capture.output(summary(comp_season,

groupNames = c("apis", "mite"),

showCentr = c("degree", "between", "closeness"),

numbNodes = 5),file="comparison.txt")
